# Large Language Models in Cardiology: Systematic Review

**DOI:** 10.2196/76734

**Published:** 2026-04-16

**Authors:** Moran Gendler, Girish N Nadkarni, Karin Sudri, Michal Cohen-Shelly, Benjamin S Glicksberg, Orly Efros, Shelly Soffer, Eyal Klang

**Affiliations:** 1 Azrieli Faculty of Medicine, Bar-Ilan University, Henrietta Szold St 8, Safed, Israel, Safed, 1311502, Israel, 972 542354444; 2 Windreich Department of AI and Human Health, Mount Sinai Medical Center, Mount Sinai, New York, NY, United States; 3 Sagol AI Hub, ARC Innovation Center, Sheba Medical Center, Ramat Gan, Israel; 4 School of Medicine, Tel Aviv University, Tel Aviv, Israel; 5 National Hemophilia Center and Thrombosis Institute, Sheba Medical Center, Ramat Gan, Israel; 6 Institute of Hematology, Davidoff Cancer Center, Rabin Medical Center, Petah-Tikva, Israel

**Keywords:** artificial intelligence, natural language processing, large language models, generative AI, LLMs, cardiology

## Abstract

**Background:**

Large language models (LLMs) are increasingly used in health care, but their role in cardiology has not yet been systematically evaluated.

**Objective:**

This review aimed to assess the applications, performance, and limitations of LLMs across diverse cardiology tasks, including chronic and progressive conditions, acute events, education, and diagnostic testing.

**Methods:**

A systematic search was conducted in PubMed and Scopus for studies published up to April 14, 2024, using keywords related to LLMs and cardiology. Studies evaluating LLM outputs in cardiology-related tasks were included. Data were extracted across 5 predefined domains and the risk of bias was assessed using an adapted QUADAS-2 tool (developed by Whiting et al at the University of Bristol). The review protocol was registered in PROSPERO (CRD42024556397).

**Results:**

A total of 33 studies contributed quantitative outcome data to a descriptive synthesis. Across chronic conditions, ChatGPT-3.5 (OpenAI) answered 91% (43/47) heart failure questions accurately, although readability often required college-level comprehension. In acute scenarios, Bing Chat omitted key myocardial infarction first aid steps in 25% (5/20) to 45% (9/20) of cases, while cardiac arrest information was rated highly (mean 4.3/5, SD 0.7) but written above recommended reading levels. In physician education tasks, ChatGPT-4 (OpenAI) demonstrated higher accuracy than ChatGPT-3.5, improving from 38% (33/88) to 66% (58/88). In patient education studies, ChatGPT-4 provided scientifically adequate explanations (5.0‐6.0/7) comparable to hospital materials but at higher reading levels (11th vs 7th grade). In diagnostic testing, ChatGPT-4 interpreted 91% (36/40) electrocardiogram vignettes correctly, significantly better than emergency physicians (31/40, 77%; *P*< .001), but showed lower performance in echocardiography.

**Conclusions:**

LLMs show meaningful potential in cardiology, especially for education and electrocardiogram interpretation, but performance varies across clinical tasks. Limitations in emergency guidance and readability, as well as small in silico study designs, highlight the need for multimodal models and prospective validation.

## Introduction

Large language models (LLMs) such as OpenAI’s ChatGPT, Google’s Gemini, and Meta’s LLaMA are advancing natural language processing by generating, understanding, and interpreting text. These models process text to produce coherent responses, understand context, summarize information, and engage in conversations [1]. Their application in health care, particularly in cardiology, offers significant benefits due to their ability to analyze diverse and complex data—from patient records to imaging studies [2,3].

In cardiology, LLMs are increasingly being used to assist in the management of cardiovascular diseases by organizing and making clinical data more accessible [4]. These models can enhance diagnostic accuracy, personalize treatment plans, and identify patterns in large datasets that traditional methods might overlook [5,6]. Additionally, LLMs offer the potential to automate routine documentation, thereby reducing the administrative burden on health care providers [7,8]. However, integrating LLMs into clinical workflows poses challenges, and effective implementation is crucial to realizing their potential to improve patient care in cardiology [8-10].

Recent reviews have highlighted the emerging role of LLMs in cardiology. Sharma et al [9] provided an early synthesis of ChatGPT applications, focusing on health literacy, clinical care, and research up to September 2023. Boonstra et al [7] more recently examined LLMs across cardiovascular disease, with emphasis on prevention and patient education. Our review complements these works by incorporating a broader search, applying PRISMA (Preferred Reporting Items for Systematic Reviews and Meta-Analyses) 2020 methodology and organizing findings into 5 clinically relevant domains. It emphasizes current uses of LLMs in cardiology, their potential impact on care and patient outcomes, and the barriers to their practical application.

## Methods

### Overview

This review was conducted according to the PRISMA guidelines [10] (Checklist 1).

### Search Strategy

A comprehensive literature search was conducted to identify studies on the application of LLMs in cardiology. The search was performed on April 14, 2024, in PubMed and Scopus, using a combination of keywords and Medical Subject Headings (MeSH) related to both cardiology and LLMs. The cardiology terms included “Echocardiography,” “Arrhythmias,” “Cardiac Output,” “Heart Failure,” “Heart Valve Diseases,” “Myocardial Ischemia,” “Acute Coronary Syndrome,” and “Electrocardiogram.” The LLM terms included “ChatGPT,” “Large Language Models,” “OpenAI,” “Microsoft Bing Chat,” “Google Bard” and “Google Gemini.” In Scopus, searches were conducted using the TITLE ABS KEY field to ensure consistency across databases. Scopus was included alongside PubMed to broaden coverage, capturing interdisciplinary studies at the intersection of artificial intelligence and cardiology that may not be indexed in PubMed. The complete search strategies are available in Multimedia Appendix 1. This review was registered with PROSPERO (CRD42024556397) [11].

### Study Selection

We included studies that (1) evaluated an application of LLMs in a specific field within cardiology, (2) were published in English, and (3) were peer reviewed. In addition to full original research articles, short reports and letters containing original data or quantitative analyses were also eligible. Studies that were non–LLM-related, non–cardiology-focused, or purely conceptual without empirical evaluation were excluded. Abstracts, conference papers, critical letters, and editorial commentaries were also excluded.

The search was supplemented by manual screening of the reference lists of included studies. Two reviewers (MG and SS) independently screened the titles and abstracts to determine whether the studies met the inclusion criteria. Full-text articles were reviewed when the title met the inclusion criteria or when there was any uncertainty. Disagreements were resolved by a third reviewer (EK).

### Data Extraction

Two independent reviewers (MG and SS) extracted data from the included studies using a standardized data extraction form. Discrepancies were resolved through discussion or consultation with a third reviewer (EK). Extracted information included study design, sample size, LLM application details (eg, LLM features examined, assessment method, validation metrics, and reference guidelines used for accuracy comparison), main findings, and limitations.

### Quality Assessment

Risk of bias was assessed using the QUADAS-2 tool (developed by Whiting et al at the University of Bristol) [12], which is widely applied in diagnostic accuracy research. This framework was selected because many included studies evaluated LLMs in diagnostic or decision-making roles, making QUADAS-2 particularly suitable for systematically assessing potential bias in study design, case selection, index test conduct, and reference standards. A detailed summary of the assessments is presented in Table S1 in Multimedia Appendix 1.

Since the studies evaluated LLM performance rather than human diagnostics, several adaptations were applied. In the patient selection domain, we assessed the transparency and representativeness of the test cases used to evaluate LLMs. Bias in this domain was considered high when studies used unreported or simplified cases that did not reflect real-world clinical variability. In the index test domain, we evaluated the standardization of prompts and scoring (number of runs and grading rules), model transparency (version, release date, and parameters), blinding to the reference standard during testing, and avoidance of post hoc prompt modification or selective reporting. The reference standard domain was adapted to assess the reliability of the comparator or ground truth (eg, expert consensus, guideline-based answers, or validated datasets).

### Data Synthesis

A narrative synthesis of the findings from the included studies was conducted. Due to anticipated heterogeneity in study designs and outcomes, a meta-analysis was not planned. Instead, the focus was on summarizing the applications, benefits, and limitations of LLMs in cardiology as reported in the included studies and identifying areas for future research. In this paper, “quantitative synthesis” refers to descriptive reporting of numerical outcomes extracted from individual studies (eg, accuracy rates, agreement statistics, readability scores, and error frequencies) without statistical pooling or calculation of combined effect estimates.

To structure the analysis, included studies were grouped into 5 categories that reflected the main areas of LLM application in cardiology. Two reviewers (MG and SS) independently categorized the studies, and any discrepancies were resolved through discussion. In cases where consensus could not be reached, a final decision was made by EK. Data were extracted for each category on study objectives, type of task, LLMs assessed, evaluation methods, and key performance outcomes.

### Chronic and Progressive Cardiac Conditions

Studies were included if they assessed LLMs in long-term cardiac conditions such as heart failure, hypertension, valvular disease, or atrial fibrillation.

#### Acute Cardiac Events

This group included studies evaluating LLMs in acute scenarios, including resuscitation, cardiac arrest, and myocardial infarction.

#### Physician Education

Studies were categorized here if they tested LLMs on cardiology training, examination-style questions, or case vignettes aimed at medical professionals. Studies that focused on physician clinical decision-making and compared it with LLM performance were also included under this group.

#### Patient Education

This group covered studies where LLMs provided information or educational content directly to patients.

#### Cardiac Diagnostics Tests

Studies were included if they examined the use of LLMs for diagnostic interpretation, such as electrocardiograms (ECGs), echocardiography, and cardiac imaging.

## Results

### Overview

**Figure 1.**
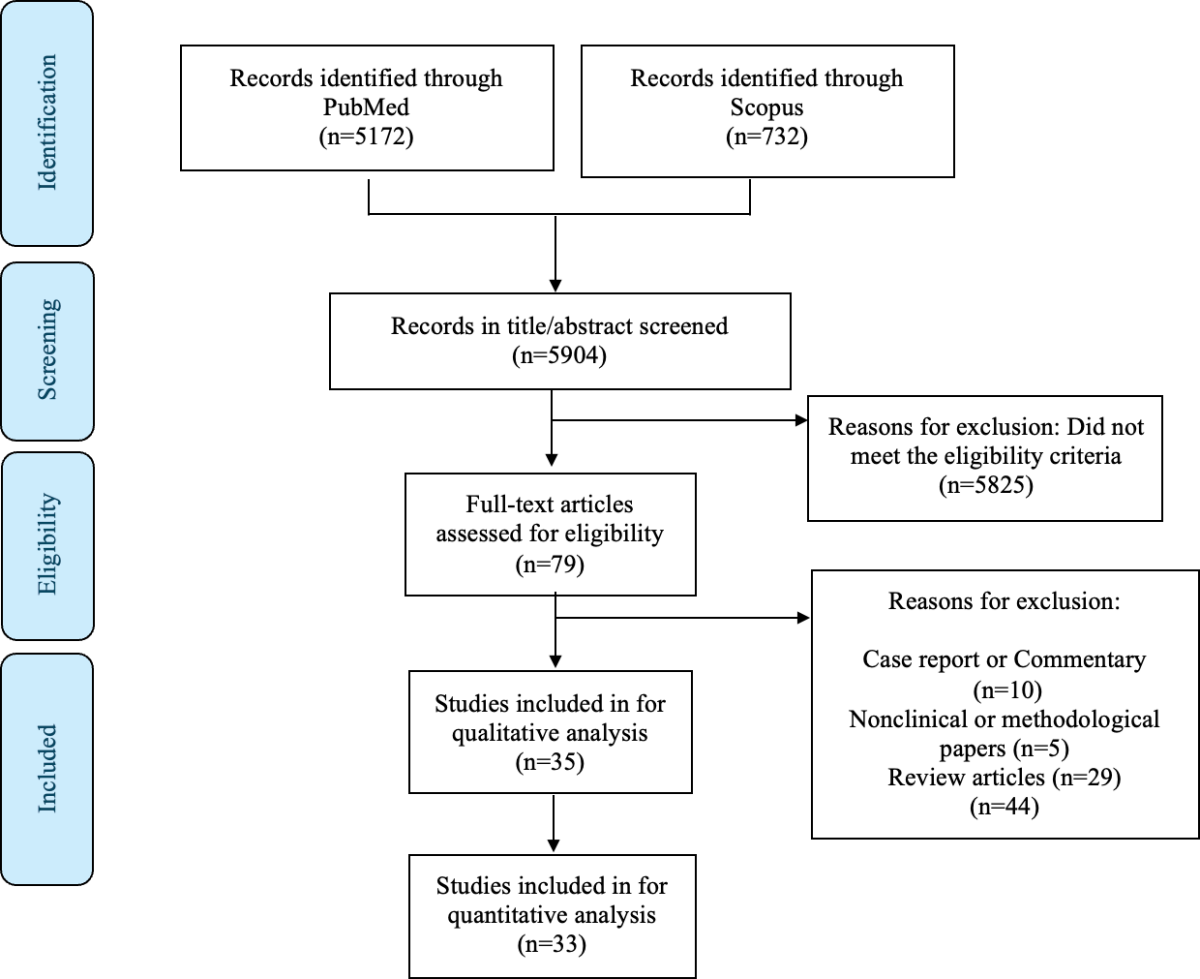
Flow diagram of the search and inclusion process.

A total of 35 articles were identified for inclusion [5,13-46]. Of these, 3 were retrieved exclusively from PubMed [27,31,39], while the remaining articles were identified in both databases. Following full-text assessment, 33 [5,13-30,33-46] studies contributed quantitative outcome data to the descriptive quantitative synthesis [5,13-30,33-46], whereas 2 studies were included in qualitative synthesis only and were therefore not included in the quantitative synthesis [31,32]. These 2 studies reported conceptual analyses and descriptive observations rather than measurable performance metrics (Figure 1).

**Figure 2.**
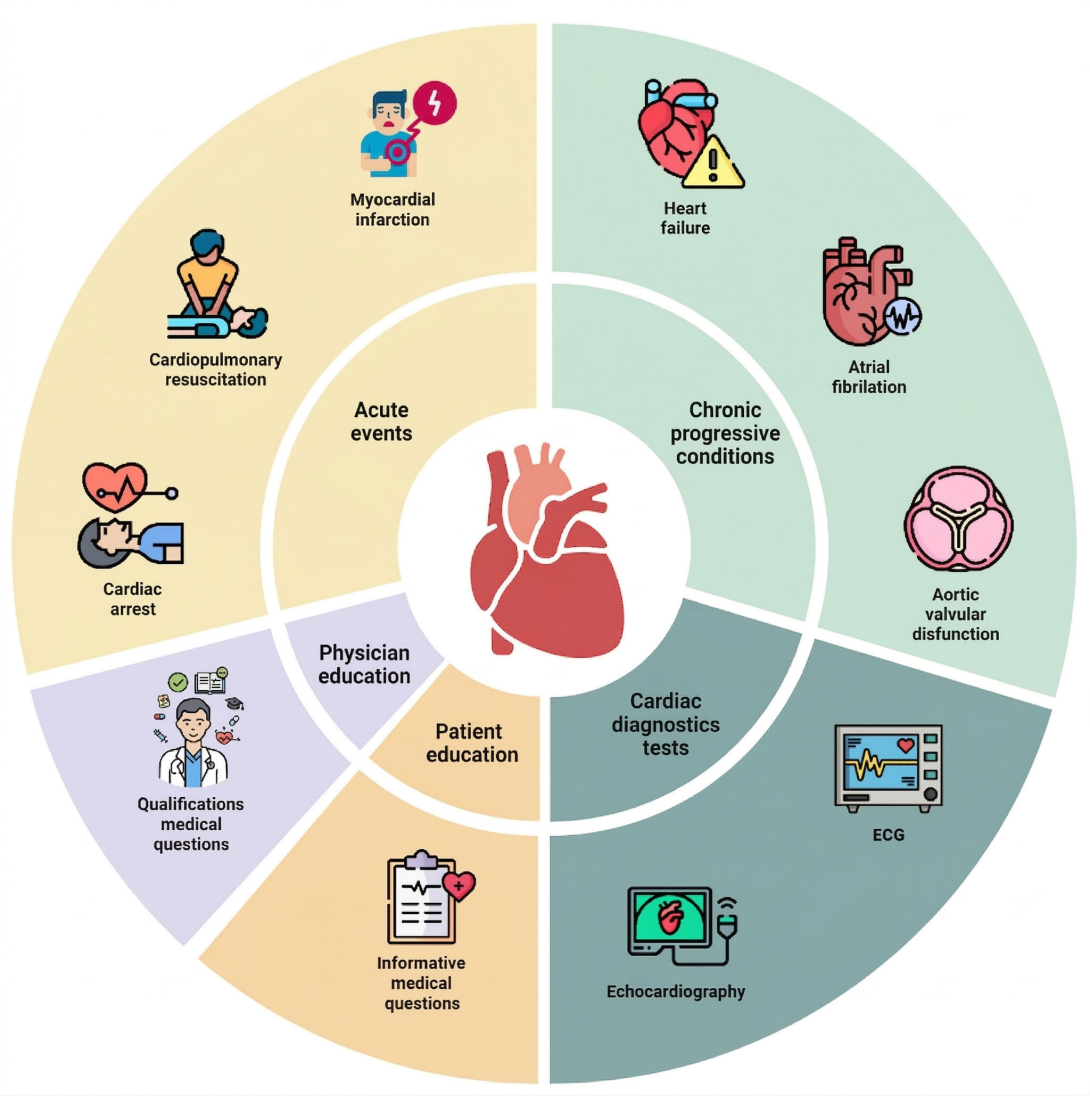
Categorization of articles into core groups with corresponding cardiology subfields.

General details about the included articles, descriptions of their characteristics, main outcomes, and their advantages and limitations are summarized in Tables1^4^, respectively. Tables S1 and S2 in Multimedia Appendix 1 provide additional detail on characteristics and outcomes. Table S3 in Multimedia Appendix 1 provides a detailed evaluation of each article using the QUADAS-2 tool. Figure 2 shows the categorization of articles into core groups with corresponding cardiology subfields.

**Table 1.** Studies included.

Category and study	Publication date	Title	Journal	PMID
Chronic and progressive cardiac conditions				
Dimitriadis et al [27]	2024 March	ChatGPT and patients with heart failure	Angiology	38451243
Riddle et al [33]	2023 December	College-level reading is required to understand ChatGPT’s answers to lay questions relating to heart failure	European Journal of Heart Failure	37964183
Krittanawong et al [34]	2023 November and December	Assessing the potential of ChatGPT for patient education in the cardiology clinic	Progress in Cardiovascular Diseases	37832625
Rouhi et al [35]	2024 March	Can artificial intelligence improve the readability of patient education materials on aortic stenosis? A pilot study	Cardiology and Therapy	38194058
Hillmann et al [36]	2023 December	Accuracy and comprehensibility of chat-based artificial intelligence for patient information on atrial fibrillation and cardiac implantable electronic devices	EP Europace	38127304
Van Bulck et al [37]	2024 January	What if your patient switches from Dr. Google to Dr. ChatGPT? A vignette-based survey of the trustworthiness, value, and danger of ChatGPT-generated responses to health questions	European Journal of Cardiovascular Nursing	37094282
Kassab et al [25]	2023 November	Comparative analysis of chat-based artificial intelligence models in addressing common and challenging valvular heart disease clinical scenarios	Journal of the American Heart Association	37982246
Han et al [24]	2024 January	Evaluation of GPT-4 for 10-year cardiovascular risk prediction- insights from the UK Biobank and KoGES^a^ data	iScience	38357664
Ali et al [23]	2023 November	Mapping the heartbeat of America with ChatGPT-4- unpacking the interplay of social vulnerability, digital literacy, and cardiovascular mortality in county residency choices	Journal of Personalized Medicine	38138852
Li et al [22]	2024 March	Potential multidisciplinary use of large language models for addressing queries in cardio-oncology	Journal of the American Heart Association	38497458
Yano et al [21]	2023 November	Relevance of ChatGPT’s responses to common hypertension-related patient inquiries	Hypertension	37916418
Kusunose et al [46]	2023 June	Evaluation of the accuracy of ChatGPT in answering clinical questions on the Japanese Society of Hypertension Guidelines	Circulation Journal	37286486
Al Tibi et al [45]	2024 March	A retrospective comparison of medication recommendations between a cardiologist and ChatGPT-4 for hypertension patients in a rural clinic	Cureus	38586651
Acute cardiac events				
Birkun and Gautam [38]	2024 January	Large language model-based chatbot as a source of advice on first aid in heart attack	Current Problems in Cardiology	37640177
Scquizzato et al [39]	2024 January	Testing ChatGPT ability to answer laypeople questions about cardiac arrest and cardiopulmonary resuscitation	Resuscitation	38081504
Safranek et al [40]	2024 March	Automated HEART^b^ score determination via ChatGPT: Honing a framework for iterative prompt development	Journal of the American College of Emergency Physicians Open	38481520
Birkun [43]	2023 August	Performance of an artificial intelligence-based chatbot when acting as EMS^c^ dispatcher in a cardiac arrest scenario	Internal and Emergency Medicine	37603142
Physician education				
Harskamp and De Clercq [41]	2024 February	Performance of ChatGPT as an AI^d^-assisted decision support tool in medicine: a proof-of-concept study for interpreting symptoms and management of common cardiac conditions (AMSTELHEART-2)	Acta Cardiologica	38348835
Skalidis and Cagnina [16]	2024 April	ChatGPT takes on the European Exam in Core Cardiology: an artificial intelligence success story?	European Heart Journal - Digital health	37265864
Yavuz and Kahraman [15]	2024 March	Evaluation of the prediagnosis and management of ChatGPT-4.0 in clinical cases in cardiology	Future Cardiol	39049771
Gritti et al [13]	2024 February	Progression of an artificial intelligence Chatbot (ChatGPT) for pediatric cardiology educational knowledge assessment	Pediatric Cardiology	38170274
Lee et al [5]	2023 October	Evaluating the clinical decision-making ability of large language models using MKSAP-19^e^ cardiology questions	JACC Advance	38938709
Patient education				
Günay et al [19]	2024 March	AI in patient education: assessing the impact of ChatGPT-4 on conveying comprehensive information about chest pain	The American Journal of Emergency Medicine	38242775
Bushuven et al [20]	2023 November	“ChatGPT, Can You Help Me Save My Child’s Life?” - diagnostic accuracy and supportive capabilities to lay rescuers by ChatGPT in prehospital basic life support and pediatric advanced life support cases - an in-silico analysis	Journal of Medical Systems	37987870
Lautrup et al [18]	2023 November	Heart-to-heart with ChatGPT: the impact of patients consulting AI for cardiovascular health advice	Open Heart	37945282
Moons and Van Bulck [17]	2024 March	Using ChatGPT and Google Bard to improve the readability of written patient information: a proof of concept.	European Journal of Cardiovascular Nursing	37603843
Almagazzachi et al [44]	2024 February	Generative artificial intelligence in patient education: ChatGPT takes on hypertension questions	Cureus	38435177
Cardiac diagnostic tests				
Fijačko et al [30]	2023 December	Can novel multimodal chatbots such as Bing Chat Enterprise, ChatGPT-4 Pro, and Google Bard correctly interpret electrocardiogram images?	Resuscitation	37884222
Zhu et al [42]	2023 July	ChatGPT can pass the AHA^f^ exams: open-ended questions outperform multiple-choice format	Resuscitation	37349064
King et al [14]	2024 February	GPT-4V passes the BLS^g^ and ACLS^h^ examinations: an analysis of GPT-4V’s image recognition capabilities	Resuscitation	38160904
Günay et al [29]	2024 March	Comparison of emergency medicine specialist, cardiologist, and chat-GPT in electrocardiography assessment	The American Journal of Emergency Medicine	38507847
Kangiszer et al [28]	2024 March	Low performance of ChatGPT on echocardiography board review questions	JACC: Cardiovascular Imaging	37943230
Sarangi et al [26]	2023 December	Radiological differential diagnoses based on cardiovascular and thoracic imaging patterns: perspectives of four large language models	The Indian Journal of Radiology & Imaging	38549881

a KoGES: Korean Genome and Epidemiology Study.

b HEART: History, ECG, Age, Risk factors, Troponin risk algorithm.

c EMS: Emergency Medical Services.

d AI: artificial intelligence.

e MKSAP-19: Medical Knowledge Self-Assessment Program (19th edition).

f AHA: American Heart Association.

g BLS: Basic Life Support.

h ACLS: Advanced Cardiovascular Life Support.

**Table 2.** Study characteristics.

Category and authors	Tasks	LLM^a^ features examined	LLM examined
Chronic and progressive cardiac conditions			
Dimitriadis et al [27]	Answering questions about the management of HF^b^.	Accuracy	ChatGPT- 3.5
Riddell et al [33]	Answer a set of hypothetical queries from a patient with HFrEF^c^.	Readability	ChatGPT- 4
Krittanawong et al [34]	Answer HF-related questions.	Reliability	ChatGPT- 3.5
Rouhi et al [35]	Rewrite patient education materials to meet recommended reading skill levels for patients with AS^d^.	Readability Simplification	Google Bard ChatGPT- 3.5
Hillmann et al [36]	Answering questions about AF^e^ and CIED^f^.	Readability Appropriateness Comprehensibility	Google Bard Bing Chat ChatGPT-4
Van Bulck and Moons [37]	Responses to virtual prompts by patients (CHD^g^, AF, HF, and Chol^h^).	Trustworthy Valuable Dangerous	ChatGPT-3
Kassab et al [25]	Answer correctly on 15 patient-centered and 15 physician-centered VHD^i^ queries.	Accuracy	ChatGPT-4 Google Bard
Han et al [24]	Predict 10-year CVD^j^ risk from cohort data.	Accuracy Robustness	ChatGPT- 3.5 ChatGPT-4
Ali et al [23]	Predicting age-adjusted cardiovascular mortality across 3118 US counties and identifying associations with social vulnerability and digital literacy indices.	Assist with regression modeling Code generation	ChatGPT-4
Li et al [22]	Answer 25 guideline-based cardio-oncology queries.	Accuracy Guideline adherence	ChatGPT- 3.5 ChatGPT-4 Google Bard Llama2 Claude2
Yano et al [21]	Answer 20 common hypertension FAQs^k^ in Japanese and English.	Appropriateness Language consistency	ChatGPT-4
Kusunose et al [46]	Answering guideline-based clinical questions on hypertension.	Accuracy	ChatGPT- 3.5
Al Tibi et al [45]	Comparing antihypertensive medication recommendations between ChatGPT-4 and a cardiologist using real -world patient data.	Accuracy	ChatGPT-4
Acute cardiac events			
Birkun and Gautam [38]	Examination of the ability of chatbots to guide first aid for heart attacks.	Guideline adherence Readability	Bing Chat
Scquizzato et al [39]	Answer lay FAQs on cardiac arrest and CPR^l^.	Accuracy Readability	ChatGPT-3.5
Safranek et al [40]	Extract data from notes and compute HEART^m^ score; test iterative prompt framework.	Accuracy Guideline adherence	ChatGPT- 3.5 ChatGPT-4
Birkun [43]	Evaluate the ability to operate as an automated assistant for recognition of cardiac arrest and real-time CPR instructions.	Accuracy Guideline adherence	New Bing chatbot
Physician education			
Harskamp and De Clercq [41]	Answering questions related to common cardiac symptoms or conditions.	Accuracy	ChatGPT- 3.5 January 2023 ChatGPT-3.5 September 2023 version
Skalidis et al [16]	Test ChatGPT on exam-style questions.	Accuracy	ChatGPT-3
Yavuz and Kahraman [15]	Assess ChatGPT-4 inthediagnosis and management of cases.	Accuracy Difficulty	ChatGPT-4
Gritti et al [13]	Compare ChatGPT-4 and ChatGPT-3.5 in accuracy on multiple-choice pediatric cardiology questions.	Accuracy	ChatGPT 3.5 ChatGPT-4
Lee et al [5]	Achieving a passing score of 50 % using MKSAP-19's^n^ cardiology questions.	Accuracy	ChatGPT- 3.5 ChatGPT-4 PubMedGPT
Patient education			
Günay et al [19]	Compare ChatGPT-4 vs hospital websites on chest pain FAQs.	Readability Guideline adherence	ChatGPT-4
Bushuven et al [20]	Evaluate ChatGPT diagnostic and capabilities in BLS^o^ and PALS^p^ scenarios.	Accuracy	ChatGPT-3.5 ChatGPT-4
Lautrup et al [18]	Respond to prompts on 4 cardiovascular topics (MI^q^, PAD^r^, VV^s^, and CP)^t^.	Accuracy	ChatGPT-4
Moons and Van Bulck [17]	Simplify patient info from journals.	Readability	ChatGPT Google Bards
Almagazzachi et al [44]	Answering hypertension questions and assessing reproducibility across repeated runs.	Accuracy	ChatGPT (version not specified)
Cardiac diagnostic tests			
Fijačko et al [30]	Interpreting ECG images.	Accuracy	Google Bard Bing Chat ChatGPT-4
Zhu et al [42]	Interpreting ECG images.	Accuracy	ChatGPT
King et al [14]	Interpreting ECG images.	Accuracy	ChatGPT-4V
Günay et al [29]	Interpreting ECG data.	Accuracy	ChatGPT-4
Kangiszer et al [28]	Answer correctly echocardiography board review questions and provide explanations that reflect standards of practice.	Accuracy	ChatGPT-4
Sarangi et al [26]	Generate DDx for 15 cardiac or thoracic imaging patterns.	Diagnostic accuracy	ChatGPT- 3.5 Google Bard Microsoft Bing Perplexity

a LLM: large language model.

b HF: heart failure.

c HFrEF: heart failure with reduced ejection fraction.

d AS: aortic stenosis.

e AF: atrial fibrillation.

f CIED: cardiac implantable electronic device.

g CHD: chronic heart disease.

h Chol: cholesterol.

i VHD: valvular heart disease.

j CVD: cardiovascular disease.

k FAQ: frequently asked question.

l CPR: cardiopulmonary resuscitation.

m HEART: History, ECG, Age, Risk factors, Troponin risk algorithm.

n MKSAP-19: Medical Knowledge Self-Assessment Program (19th edition).

o BLS: Basic Life Support.

p PALS: Pediatric Advanced Life Support.

q MI: myocardial infarction.

r PAD: peripheral arterial disease.

s VV: varicose veins.

t CP: cardiovascular prevention.

**Table 3.** Outcomes of large language model applications.

Group and manuscript	Quantitative
Chronic and progressive cardiac conditions	
Dimitriadis et al [27]	ChatGPT-3.5 answered 43/47 (91%) HF^a^ patient questions adequately; 4/47 (9%) right but insufficient.
Riddell et al [33]	ChatGPT-4 responses to FAQs^b^: 71% (50/70, median FRE^c^ 40.2, grade 16, IQR 48.3-34.6) at college-level readability; 23% (16/70) at recommended lower than college level and 4% (4/70) of responses requiring grade 8‐9.
Krittanawong et al [34]	On 20 HF patient questions, ChatGPT was reliable with explanation in 40% (8/20), reliable without explanation in 40% (8/20), and unreliable in 20% (4/20).
Rouhi et al [35]	ChatGPT-3.5 simplified aortic stenosis materials to 6th-7th grade, Bard to 8th-9th; both improved from college-level baseline, all *P*<.001.
Hillmann et al [36]	On 25 AF^d^ questions, ChatGPT-4 produced 84% (21/25) appropriate and 92% (23/25) comprehensible responses with 24% (6/25) missing content. On 25 CIED^e^ questions, ChatGPT-4 produced 88% (22/25) appropriate and 100% (25/25) comprehensible responses with 52% (13/25) missing content.
Van Bulck and Moons [37]	40% (8/20) of experts rated ChatGPT’s information as more valuable than Google, 45% (9/20) as equally valuable, and 15% (3/20) as less valuable.
Kassab et al [25]	ChatGPT-4 provided 100% (15/15) accurate responses to patient-centered questions and 73% (11/15) accurate with 27% (4/15) partly accurate responses to complex clinical scenarios, outperforming Google Bard (40% [6/15] accurate).
Han et al [24]	ChatGPT-4 achieved AUROC^f^ 0.725 in the UK Biobank and 0.664 in the KoGES^g^ cohort for 10-year CVD^h^ risk prediction, performing comparably to the ACC^i^ or AHA^j^ (0.733, 0.674) and Framingham (0.728, 0.675) models.
Ali et al [23]	ChatGPT-4–assisted regression explained 34% (R²=0.34) of the variability in age-adjusted cardiovascular mortality, with higher social vulnerability increasing mortality (β=+49.01) and greater digital literacy reducing it (β=–4.51).
Li et al [22]	On 25 cardio-oncology questions, ChatGPT-4 provided 68% (17/25) appropriate responses, followed by Bard, Claude 2, and ChatGPT-3.5 with 52% (13/25), and Llama 2 with 48% (12/25) (*P*=.65).
Yano et al [21]	ChatGPT-4’s responses were rated appropriate in 85% (17/20) of cases, with strong interreviewer agreement (Gwet AC=0.890, SE 0.066, *P*<.001).
Kusunose et al [46]	Overall accuracy 64.5% (20/31). Accuracy was higher for clinical questions (CQs) than for limited evidence-based questions: 80% (16/20) vs 36% (4/11) (*P*=.005). Across 21 CQs, 9 showed zero entropy (identical answers), while 7 of the remaining 12 had entropy >0.5 (unacceptable variability).
Al Tibi et al [45]	Overall recommendations conflicted in 95% (38/40)**;** Cohen κ=−0.0127 (no agreement). Category match: stop 0%**,** decrease 0%**,** increase 6.7% (3/40)**,** add 12.5% (5/40).
Acute cardiac events	
Birkun and Gautam [38]	In 60 Bing chatbot responses, inconsistent advice appeared in 25% (5/20) of responses for the Gambia and the United States and 45% (9/20) for India. Readability required a 12th-grade level for the Gambia and the United States and 10th grade for India (*P*≤.008).
Scquizzato et al [39]	ChatGPT-3.5 answers to cardiac arrest and CPR^k^ questions were rated positively overall (mean 4.3/5, SD 0.7), with high scores for clarity (mean 4.4/5, SD 0.6), relevance (mean 4.3/5, SD 0.6), and accuracy (mean 4.0/5, SD 0.6).
Safranek et al [40]	ChatGPT-4 reduced nonnumerical errors from 5.7% (95% CI 3.6‐8.9) to 0.3% (0.1‐1.9), lowered subscore error to 0.10 (0.07‐0.14) points with less variability (SD 0.33), and correctly classified HEART^l^ risk groups in 100% (96.3‐100) of runs, compared with 81.5% (71.7‐88.4) for ChatGPT-3.5.
Birkun [43]	In Scenario 1, the chatbot suggested inapplicable or excessive actions in 10% (1/10) of conversations; in Scenario 2, this occurred in 30% (3/10). In Scenario 2, the chatbot failed to transition to CPR instructions after assessing the victim’s condition in 30% (3/10).
Physician education	
Harskamp and De Clercq [41]	The January 2023 version of ChatGPT-3.5 performed significantly worse, answering 74% (37/50) versus 92% (46/50) of trivia questions compared with the September 2023 ChatGPT version (*P*=.03), and only 50% (10/20) of complex cases were answered correctly.
Skalidis et al [16]	ChatGPT answered 58.8% (213/362) correctly. ESC^m^ 61.7% (42/68), BHDRA^n^ 52.6% (79/150), StudyPRN 63.8% (92/144), approximating the 60% passing threshold.
Yavuz and Kahraman [15]	ChatGPT-4 received high expert agreement for differential diagnoses (median 5, IQR 1) and management plans (median 4, IQR 1), with diagnostic accuracy of 4.47, SD 0.81 in Group 1 and 4.58, SD 0.67 in Group 2, with no significant difference between groups (*P*<.26).
Gritti et al [13]	ChatGPT-4 answered 66% (58/88) correctly, significantly outperforming ChatGPT-3.5 at 38% (33/88); *P*<.001, with superior accuracy across every subspecialty topic.
Lee et al [5]	ChatGPT-4 outperformed average MKSAP-19^o^ users 80% (96/120) versus 60% (72/120); *P*<.001; ChatGPT-3.5 also passed but lower at 55% (66/120), while PubMedGPT failed at 27% (32/120).
Patient education	
Günay et al [19]	Readability analysis showed: hospital website answers averaged a Flesch Reading Ease score of 65.6 (7th grade level), whereas ChatGPT-4 responses averaged 43.3 (11th grade level).
Bushuven et al [20]	ChatGPT-3.5 and ChatGPT-4 correctly identified the diagnosis in 94% (124/132; *P*=.49) of responses, but advised emergency calls in only 54% (12/22) and provided correct first aid guidance in 45% (10/22), with incorrect advanced life support instructions in 14% (3/22) of cases.
Lautrup et al [18]	ChatGPT-4 responses to 123 cardiovascular prompts averaged 3‐4 across the 4Cs. Myocardial infarction prompts scored highest (correctness 3.84/5; conciseness 3.65/5), while cardiovascular prevention scored lowest (correctness 3.03/5; conciseness 2.71/5).
Moons and Van Bulck [17]	ChatGPT lowered readability modestly (JAMA grade 11→9; Cochrane 17→11; EJCN grade 10 unchanged) while preserving most content, with word counts changing minimally in JAMA (533→525), by 14% in Cochrane (365→315), and by 45% in EJCN (1,013→563).
Almagazzachi et al [44]	Appropriateness: 93% (93/100) overall and 7% (7/100) inappropriate, evaluated against guideline-based standards. Reproducibility: 93% (93/100) of questions reproducible and 7% (7/100) irreproducible.
Cardiac diagnostic tests	
Fijačko et al [30]	ChatGPT-4 was correct in 17/27 (63%), Bard 13/27 (48.2%), and Bing 6/27 (22.2%).
Zhu et al [42]	ChatGPT achieved 84% (21/25) overall accuracy on BLS^p^ and 78.9% (30/38) on evaluable ACLS^q^ items using multiple-choice inputs, improving to 96% (24/25) and 92.1% (35/38) when incorrectly answered questions were rewritten as open-ended prompts.
King et al [14]	ChatGPT-4V answered 96%(24/25) BLS and 90% (45/50) ACLS questions correctly, accuracy decreased to 75% (9/12) for questions containing ECG^r^.
Günay et al [29]	ChatGPT-4 correctly answered 91% (36/40), outperforming emergency medicine specialists 77% (31/40, *P*<.001) and cardiologists 82% (33/40, *P*=.001).
Kangiszer et al [28]	ChatGPT-4 answered 47% (67/141) in open-ended format, 53% (75/141) in multiple choice without justification, and 55% (78/141) in multiple choice with justification formats correctly.
Sarangi et al [26]	Perplexity performed highest with 67% (50/75) concordance, followed by ChatGPT at 65% (49/75) and Bing at 63% (47/75), while Bard showed the lowest performance at 45% (34/75).

a HF: heart failure.

b FAQ: frequently asked question.

c FRE: Flesch Reading Ease.

d AF: atrial fibrillation.

e CIED: cardiac implantable electronic device.

f AUROC: area under the receiver operating characteristic curve.

g KoGES: Korean Genome and Epidemiology Study.

h CVD: cardiovascular disease.

i ACC: American College of Cardiology.

j AHA: American Heart Association.

k CPR: cardiopulmonary resuscitation.

l HEART: History, ECG, Age, Risk factors, Troponin risk algorithm.

m ESC: European Society of Cardiology.

n BHDRA: British Heart Data Research Alliance.

o MKSAP-19: Medical Knowledge Self-Assessment Program (19th edition).

p BLS: Basic Life Support.

q ACLS: Advanced Cardiovascular Life Support.

r ECG: electrocardiogram.

**Table 4.** Strengths and limitations.

Group and study	LLM^a^ advantages	LLM disadvantages and limitations	Conclusion
Chronic and progressive cardiac conditions			
Dimitriadis et al [27]	Gave clear and supportive answers to common HF^b^ questions.	Using a single LLM version and a fixed question source; lacked real patient interaction, no quantitative scoring.	Useful for patient education in HF, but the evidence is limited.
Riddell et al [33]	Accurate and consistent responses; objective readability assessment using validated metrics (FRE^c^ and SMOG^d^).	Questions were not validated with real patients; responses were tested on a single model sample.	Reliable content, but readability should be improved for patient use.
Krittanawong et al [34]	ChatGPT model provided primarily reliable answers to commonly asked questions related to HF.	Prompts not validated with real patients; single LLM tested; lacked quantitative scoring and external validation.	Promising adjunct for HF education, not stand-alone.
Rouhi et al [35]	Used standardized readability metrics on PEMs^e^ from major institutions; compared the LLMs under identical conditions.	Focused on readability only; small sample of 21 materials; limited to US sources.	Both LLMs improved the readability of aortic stenosis materials, but broader validation and comprehension testing are needed before patient application.
Hillmann et al [36]	Standardized comparison across 3 LLMs using expert-blinded evaluation.	Questions not externally validated; small dataset (50 items); limited to electrophysiology topics.	ChatGPT-4 outperformed Bing and Bard in accuracy and comprehensibility, showing strong potential for patient education.
Van Bulck and Moons [37]	ChatGPT provided clearer, more structured, and more reliable cardiology information than Google.	Very small sample (4 vignettes and 20 experts); prompts not validated with real patients.	ChatGPT is generally seen as trustworthy and useful, but evidence remains limited.
Kassab et al [25]	ChatGPT-4 is highly accurate for patient and physician queries; it outperformed Bard.	Small dataset (15 patient and 15 physician questions); subjective grading.	ChatGPT-4 is promising for patient education and clinician support in valvular disease.
Han et al [24]	Large, real-world cohorts (UK Biobank and KoGES^f^) with transparent methodology; ChatGPT-4 achieved accuracy comparable to established CVD^g^ risk models.	ChatGPT-4 outputs vary with identical prompts; training data remain non transparent.	ChatGPT-4 is feasible for population-level CVD risk prediction.
Ali et al [23]	Innovative use of ChatGPT-4 for epidemiology.	Reliance on secondary data sources may introduce reporting bias; ChatGPT-4’s role is limited to regression assistance.	LLMs may complement population health research, but clinical relevance is limited.
Li et al [22]	Compared multiple LLMs using standardized ESC^h^ guideline–based cardio-oncology questions.	Small question set, questions researcher-generated rather than patient-derived.	ChatGPT-4 shows promise for cardio-oncology, requires oversight.
Yano et al [21]	First study to evaluate ChatGPT-4’s hypertension responses in English and Japanese.	Small sample (20 questions); prompts generated by the model rather than real patients; subjective evaluation.	ChatGPT-4 provided accurate and guideline-consistent hypertension information in both languages.
Kusunose et al [46]	Potential supplementary tool for rapid access to hypertension guideline information.	Overall accuracy may be insufficient for standalone use; inconsistent answers on repeat runs (entropy); small sample; single grading evaluation; no assessment of downstream clinical outcomes.	ChatGPT may assist clinicians as a supplement, but requires caution, especially for complex questions.
Al Tibi et al [45]	Uses real-world patient data.	Single center and single cardiologist; assumes physician is correct; limited context given to ChatGPT-4; small sample.	ChatGPT-4 recommendations differed substantially from those of the cardiologist, with no agreement; further validation is needed before clinical use.
Acute cardiac events			
Birkum and Gautam [38]	Using repeated queries across countries.	Single nonvalidated prompt; frequent omissions of key guideline steps.	Provides relevant but often incomplete or incorrect MI^i^ first aid advice, limited for unsupervised public use.
Scquizzato et al [39]	Dual evaluation by professionals and laypeople; generally positive ratings.	High reading level; subjective, unblinded scoring; single LLM version.	ChatGPT provided useful and mostly accurate CPR^j^ information, but readability and safety gaps limit unsupervised use.
Safranek et al [40]	The framework prompts improvement for automated HEART^k^ score determination across a limited set of synthetic patient notes.	Synthetic dataset; HEART subscores limited to structured fields.	Promising clinician decision support concept that warrants validation on real clinical data.
Birkun [43]	Natural dialogue; frequently delivered straightforward CPR steps and handled a bystander barrier by encouraging continuation.	Omitted critical elements; diagnostic risk by not asking “breathing normally”; conversational glitches and occasional inapplicable suggestions.	May be a better-than-nothing option where T-CPR^l^ is unavailable, but it should not be considered reliable for real-life emergencies.
Physician education			
Harskamp and De Clercq [41]	Clear performance improvement in newer model versions.	Lower accuracy in complex consults; single run prompts; limited model transparency.	ChatGPT showed potential as AI^m^ decision support for common cardiac conditions but requires further validation before clinical adoption.
Skalidis et al [16]	Large exam dataset; transparent question sourcing.	Manual single run prompting; no control of model settings.	Potential aid for exam prep.
Yavuz and Kahraman [15]	High expert agreement for differential diagnoses.	Synthetic case format may not reflect real-world nuance; modest variability in expert ratings.	Useful adjunct for training, but structured oversight is needed.
Gritti et al [13]	ChatGPT-4 markedly improved accuracy over ChatGPT-3.5; outperformed across all subspecialties; objective scoring using textbook answer key.	Moderate overall accuracy; text-only questions (no ECG or echo); single-run testing.	ChatGPT-4 performs better than ChatGPT-3.5 but remains insufficient for high-stakes pediatric cardiology use.
Lee et al [5]	Uses validated MKSAP-19 answer key.	Exam style questions only (no ECG, echo, or images); single run manual prompting.	ChatGPT-4 shows strong decision support potential, but limitations must be managed.
Patient education			
Günay et al [19]	Direct comparison with hospital websites; blinded expert rating.	Language complexity; no patient validation.	Scientifically sound but limited accessibility
Bushuven et al [20]	High diagnostic accuracy (94%) and reliable recognition of pediatric emergencies.	Simulated vignettes only; no direct comparison with humans’ competence in emergency situations.	ChatGPT-4 performs better than ChatGPT-3.5 in emergency recognition but still provides incorrect and inconsistent guidance, requiring further refinement before real-world use.
Lautrup et al [18]	Innovative 4C framework; diverse prompts.	Expert-based, not real patients; no replicate testing; no control of model parameters.	Useful framework, but highlights risks of LLM-driven patient advice.
Moons and Van Bulck [17]	Clear value of LLMs for simplifying patient information and improving readability.	ChatGPT rarely reaches 6th-grade level; Bard removes large amounts of text; English-only evaluation; visuals not assessed; temperature settings not tested.	ChatGPT is useful for simplifying Patient Education Materials, but requires further evaluation.
Almagazzachi et al [44]	Large curated question set; repeated queries to assess reproducibility; dual evaluation against guidelines and physician judgment.	Predefined questions may limit topic coverage; no patient user testing; model version not specified.	ChatGPT demonstrated high accuracy and reproducibility for hypertension patient education, but human oversight remains necessary.
Cardiac diagnostic tests			
Fijačko et al [30]	First multimodal chatbot ECG^n^ test.	Small dataset	Proof of concept multimodal LLMs can attempt ECG.
Zhu et al [42]	Open-ended prompts improved answer quality.	Open-ended prompts improved answer quality; evaluation restricted to exam-style items rather than real-world clinical variation.	ChatGPT can achieve high performance on AHA^o^ exam content, especially when questions are reframed as open-ended prompts.
King et al [14]	ChatGPT-4V outperformed GPT-3.5, particularly with the inclusion of image-based questions.	No prospective testing with residents; only multiple-choice focus.	GPT-4 may support test preparation and training, but is limited to narrow tasks.
Günay et al [29]	ChatGPT showed better accuracy than the other 2 groups in everyday ECG questions.	Used text, not real ECG images; possible training exposure.	ChatGPT-4 is strong, but not a replacement.
Kangiszer et al [28]	Consistent strength in fact-based questions .	Low overall accuracy; no image interpretation capability evaluated; no comparison to human trainees.	ChatGPT-4 shows limited accuracy for echocardiography board content.
Sarangi et al [26]	Ability to generate reasonable differential diagnoses from text prompts.	Only text descriptions; 2 radiologists only.	Useful adjunct, but true concordance with experts remains limited.

a LLM: large language model.

b HF: heart failure.

c FRE: Flesch Reading Ease.

d SMOG: Simple Measure of Gobbledygook.

e PEM: Patient Education Materials.

f KoGES: Korean Genome and Epidemiology Study.

g CVD: cardiovascular disease.

h ESC: European Society of Cardiology.

i MI: myocardial infarction.

j CPR: cardiopulmonary resuscitation.

k HEART: History, ECG, Age, Risk factors, Troponin risk algorithm.

l T-CPR: telecommunicator-assisted cardiopulmonary resuscitation.

m AI: artificial intelligence.

n ECG: electrocardiogram.

o AHA: American Heart Association.

### Chronic and Progressive Cardiac Conditions

Thirteen studies [21-25,27,33-37,45,46] evaluated the application of LLMs in chronic cardiovascular disease, spanning heart failure, hypertension, valvular disease, atrial fibrillation, cardiovascular risk prediction, and cardio-oncology.

Heart failure was the most frequently studied topic. Dimitriadis et al [27] showed that ChatGPT-3.5 produced accurate answers to 91% (43/47) of patient questions, though some were incomplete. Riddell et al [33] reported that ChatGPT-4 responses to HF questions were written at a college reading level in 71% (50/70; median FRE [Flesch Reading Ease] 40.2, grade 16, IQR 48.3-34.6) of cases, while Krittanawong et al [34] reported that only 40% (8/20) of ChatGPT-3.5 responses were reliable without physician oversight.

Hypertension was addressed by Yano et al [21], who demonstrated that ChatGPT-4 produced largely appropriate answers in 85% (17/20) of hypertension-related inquiries in English and Japanese, with English responses consistently superior. Kusunose et al [46] evaluated ChatGPT-3.5 against 31 guideline-based questions derived from the Japanese Society of Hypertension (JSH) 2019 guidelines. The chatbot achieved an overall accuracy of 64.5% (20/31). Performance was significantly higher for clinical questions than for limited evidence-based questions (80% [16/20] vs 36% [4/11]; *P*=.005). A nonsignificant trend was observed for recommendation level versus evidence level questions (62% vs 38%; denominators not reported; *P*=.07). No difference was found between questions originally written in Japanese and translated questions (65% vs 58%; denominators not reported; *P*=.60). In a retrospective analysis using real-world data from a rural clinic, Al Tibi et al [45] compared antihypertensive medication recommendations generated by ChatGPT-4 with those made by a cardiologist during laboratory review visit. Among 40 patients with hypertension, overall recommendations differed in 95% (38/40) of cases. At the level of individual medications, agreement was low, with only 10.2% of recommendations matching between ChatGPT-4 and the cardiologist (denominators not reported). The Cohen κ coefficient was −0.0127, indicating no agreement on whether to implement medication changes for a given patient.

Valvular disease was the focus of Rouhi et al [35], who showed ChatGPT-3.5 and Bard improved readability of aortic stenosis education materials, with ChatGPT-3.5 achieving the target 6th-7th grade level while Bard remained above it. Additionally, Kassab et al [25] evaluated 30 valvular disease queries, reporting ChatGPT-4 provided 100% (15/15) accurate responses to patient-centered questions and 73% (11/15) accurate and 27% (4/15) partly accurate responses to complex clinical scenarios, outperforming Google Bard (40% [6/15] accurate) and being 2.5-fold more likely to provide accurate answers (*P*<.001).

Atrial fibrillation and cardiac implantable device information were evaluated by Hillmann et al [36]; ChatGPT-4 produced appropriate responses in 84% (21/25) of atrial fibrillation and 88% (22/25) of cardiac implantable electronic device queries, with comprehensibility scores of 92% (23/25) and 100% (25/25), respectively. ChatGPT-4 outperformed Bing (60% [15/22], 72% [18/25] appropriate) and Bard (52% [13/25], 16% [4/25] appropriate) and showed fewer omissions and minimal confabulation.

Other chronic conditions were also explored. Han et al [24] showed ChatGPT-4 achieved 10-year cardiovascular disease risk predictions with performance similar to American College of Cardiology/American Heart Association (AHA). Li et al [22] found ChatGPT-4 outperformed other LLMs in cardio-oncology, though it was less reliable for treatment recommendations. Van Bulck and Moons [37] reported that 40% (8/20) of experts found ChatGPT’s information more valuable than Google, 45% (9/20) equally valuable, and 15% (3/20) less valuable. Experts appreciated the sophistication and nuance of ChatGPT’s responses but noted they were sometimes incomplete or potentially misleading.

Cardiovascular mortality was evaluated by Ali et al [23], who demonstrated the use of ChatGPT-4 to generate and execute regression models predicting mortality rates for the US county level. Across 3118 counties, the model explains 34% (*R*²=0.34) of variability in age-adjusted cardiovascular mortality with higher social vulnerability increasing (*β*=49.01) and higher digital literacy reducing (β =−4.51) mortality.

### Acute Cardiac Events

Four studies [38-40,43] evaluated LLMs in acute cardiac contexts.

#### First Aid in Myocardial Infarction

Birkun and Gautam [38] found that the Bing chatbot frequently omitted critical guideline concordant steps, with readability of 12th grade level for the Gambia and the United States and 10th grade for India (*P*≤.008). Incorrect advice appeared in 25% (5/20) of responses for the Gambia and the United States and 45% (9/20) for India.

#### Cardiac Arrest and Cardiopulmonary Resuscitation

Scquizzato et al [39] reported that ChatGPT-3.5 answers to cardiac arrest and cardiopulmonary resuscitation (CPR) questions were rated positively overall (mean 4.3/5, SD 0.7), with high scores for clarity (mean 4.4/5, SD 0.6), relevance (mean 4.3/5, SD 0.6), and accuracy (mean 4.0/5, SD 0.6). CPR-specific responses scored lower across all parameters, and professionals rated overall value (mean 4.0/5, SD 0.5 vs mean 4.6/5, SD 0.7; *P*=.02) and comprehensiveness (mean 3.9/5, SD 0.6 vs mean 4.5/5, SD 0.7; *P*=.02) lower than laypeople. Readability was difficult (FRE score 34 [IQR 26‐42]). Birkun [43] assessed the New Bing chatbot’s ability to provide telecommunicator-assisted CPR across 2 scenarios: Scenario 1, in which the victim was not breathing, and Scenario 2, in which the bystander was unsure whether the victim was breathing. In Scenario 2, the chatbot failed to ask for the emergency address in 50% (5/10) of cases and did not transition to CPR instructions after assessing the victim in 30% (3/10), with several additional Scenario 2 conversations reportedly interrupted or stuck at the breathing assessment step. The chatbot asked only whether the victim was “breathing” (rather than “breathing normally”), potentially missing agonal breathing and delaying arrest recognition, never inquired about nearby AED (Automated External Defibrillator) availability, and suggested inapplicable actions in 10% (1/10) of Scenario 1 and 30% (3/10) of Scenario 2.

#### Chest Pain Evaluation

Safranek et al [40] reported ChatGPT-4 correctly classified HEART score (History, ECG, Age, Risk factors, Troponin risk algorithm) risk groups in 100% (96.3%‐100%) of runs compared with 81.5% (71.7%‐88.4%) for ChatGPT-3.5. Iterative prompt refinement reduced nonnumerical outputs for ChatGPT-3.5 from 18.7% (95% CI 14.7‐23.5) to 6.7% (4.4‐10.1) and for ChatGPT-4 from 5.7% (3.6‐8.9) to 0.3% (0.1‐1.9).

### Physician Education

Six studies [5,13,15,16,19,41] investigated the use of LLMs for supporting physician training and assessment in cardiology.

Exam preparation was assessed by Lee et al [5], who tested LLMs on 120 MKSAP-19 (Medical Knowledge Self-Assessment Program, 19th edition) cardiology questions, found that ChatGPT-4 achieved 80% (96/120), meeting the passing threshold, while PubMedGPT lagged far behind at 27% (32/120). Skalidis et al [16] reported that ChatGPT answered 58.8% (213/362) of European Exam in Core Cardiology questions correctly, close to the 60% passing threshold.

Clinical cases were addressed by Yavuz and Kahraman [15], who reported that ChatGPT-4 achieved high expert agreement for differential diagnoses (median 5, IQR 1) and management plans (median 4, IQR 1), supporting its role as a supplemental study aid, but emphasized that it should not be used unsupervised.

Clinical reasoning and decision support were tested by Harskamp and De Clercq [41]. ChatGPT-3.5 achieved correct responses in 85% (17/20) of AMSTELHEART-2 case vignettes, though performance was inconsistent in complex presentations. Gritti et al [13] found ChatGPT-4 correctly answered 66% (58/88) of pediatric cardiology cases, compared with 38% (33/88; *P*<.001) for ChatGPT-3.5, with superior accuracy across all subspecialty topics, when the passing threshold was set at 70%. Both models produced explanations containing incorrect or inconsistent reasoning, which were not formally graded.

Chest pain information was assessed by Günay et al [19], who found that ChatGPT-4 produced answers with comparable scientific adequacy, ease of understanding, and physician satisfaction to hospital websites (all 5.0‐6.0/7; no significant differences), but at a much higher reading level—11th grade versus 7th grade for hospital materials.

### Patient Education

Four studies [17-20] evaluated LLMs for patient information. General consultation with ChatGPT-4 was explored by Lautrup et al [18], where its responses to 123 cardiovascular prompts scored between 3 and 4 across the 4Cs (correctness 3.45/5, conciseness 3.19/5, comprehensiveness 3.52/5, and comprehensibility 3.72/5). Performance varied by topic, with myocardial infarction prompts scoring highest (correctness 3.84/5 and conciseness 3.65/5) and cardiovascular prevention lowest (correctness 3.03/5 and conciseness 2.71/5). Higher literacy prompts yielded better responses, while lower resource language prompts unexpectedly scored higher across all domains.

Emergency situations were studied by Bushuven et al [20], who compared ChatGPT-3.5 and ChatGPT-4 in Basic Life Support (BLS) and Pediatric Advanced Life Support (PALS) cases. While both models correctly identified the diagnosis in 94% (124/132; *P*=.49) of cases, they advised calling emergency services in only 54% (12/22), provided correct first aid guidance in 45% (10/22), and gave incorrect advanced life support instructions in 14% (3/22) of cases.

Readability of patient materials was evaluated by Moons and Van Bulck [17], who found that ChatGPT improved readability with minimal content loss (JAMA 533 to 525 words; Cochrane 365 to 315 words; EJCN 1013 to 563 words), whereas Google Bard achieved lower grade levels, but removed substantial content—shortening the texts by 61% (525 to 207 words), 34% (365 to 242 words), and 80% (1013 to 204 words), often omitting important details.

Almagazzachi et al [44] compiled a final set of 100 hypertension-related questions after physician review. Each question was asked to ChatGPT 3 times, and the majority response for each question was evaluated against established reference publications. Guideline-based assessment classified 93% (93/100) of the majority of responses as appropriate and 7% (7/100) as inappropriate. A separate clinical review by 1 board-certified internal medicine physician classified 92% (92/100) as appropriate and 8% (8/100) as inappropriate, yielding an overall accuracy of 92.5% (mean of the 2 assessments). For reproducibility per question, 93% (93/100) were reproducible and 7% (7/100) were irreproducible; across all 300 responses, 3.6% (7/300) were classified as irreproducible.

### Cardiac Diagnostics Tests

Six studies examined the ability of LLMs to support diagnostic testing in cardiology [14,26,28-30,42], focusing on imaging, electrocardiography, and echocardiography.

Cardiothoracic imaging was evaluated by Sarangi et al [26], who compared 4 LLMs on 25 cardiac differential diagnosis items. ChatGPT and Perplexity provided more consistent differential diagnoses than Bing and Bard (67% [50/75] vs Bing 63% [47/75] and Bard 45% [34/75]), though accuracy remained moderate and dependent on case complexity.

Electrocardiography performance was assessed across several studies. Fijačko et al [30] evaluated multimodal LLMs on ECG image interpretation, finding that ChatGPT-4 Pro correctly interpreted 63% (17/27) of ECG images, outperforming Google Bard 48% (13/27) and Bing 22.2% (6/27). Zhu et al [42] subsequently assessed ChatGPT-4 on AHA BLS or ACLS (Advanced Cardiovascular Life Support) examination items. Although the model achieved 84% (21/25) accuracy on BLS items and 78.9% (30/38) on evaluable ACLS questions using multiple choice prompts, the majority of its errors originated from ECG-containing items. Accuracy improved substantially to 96% (24/25) for BLS and 92.1% (35/38) for ACLS, when incorrectly answered multiple-choice questions were rewritten as open-ended prompts. More recently, King et al [14] evaluated ChatGPT-4V on the full 75-item AHA BLS or ACLS examination, achieving 96% (24/25) accuracy on BLS and 90 % (45/50) on ACLS items, with performance decreasing to 75% (9/12) on questions that contained ECG strips in the ACLS examination.

In a separate vignette-based evaluation, Günay et al [29] tested ChatGPT-4 on 40 written ECG case scenarios, finding 91% (36/40) accuracy exceeding that of emergency physicians 77% (31/40; *P*<.001) and comparable to cardiologists 82% (33/40; *P*=.001), though the model consistently struggled with wide QRS tachycardias.

Echocardiography was evaluated by Kangiszer et al [28], who tested ChatGPT-4 on 150 echocardiography board-style questions, answered 141. Accuracy remained modest, with ChatGPT-4 correctly answering 47.3% (67/141) of open-ended items, 53.3% (75/141) of multiple-choice items without justification, and 55.3% (78/141) with forced justification. Overall performance was inadequate for board-level competency.

**Figure 3.**
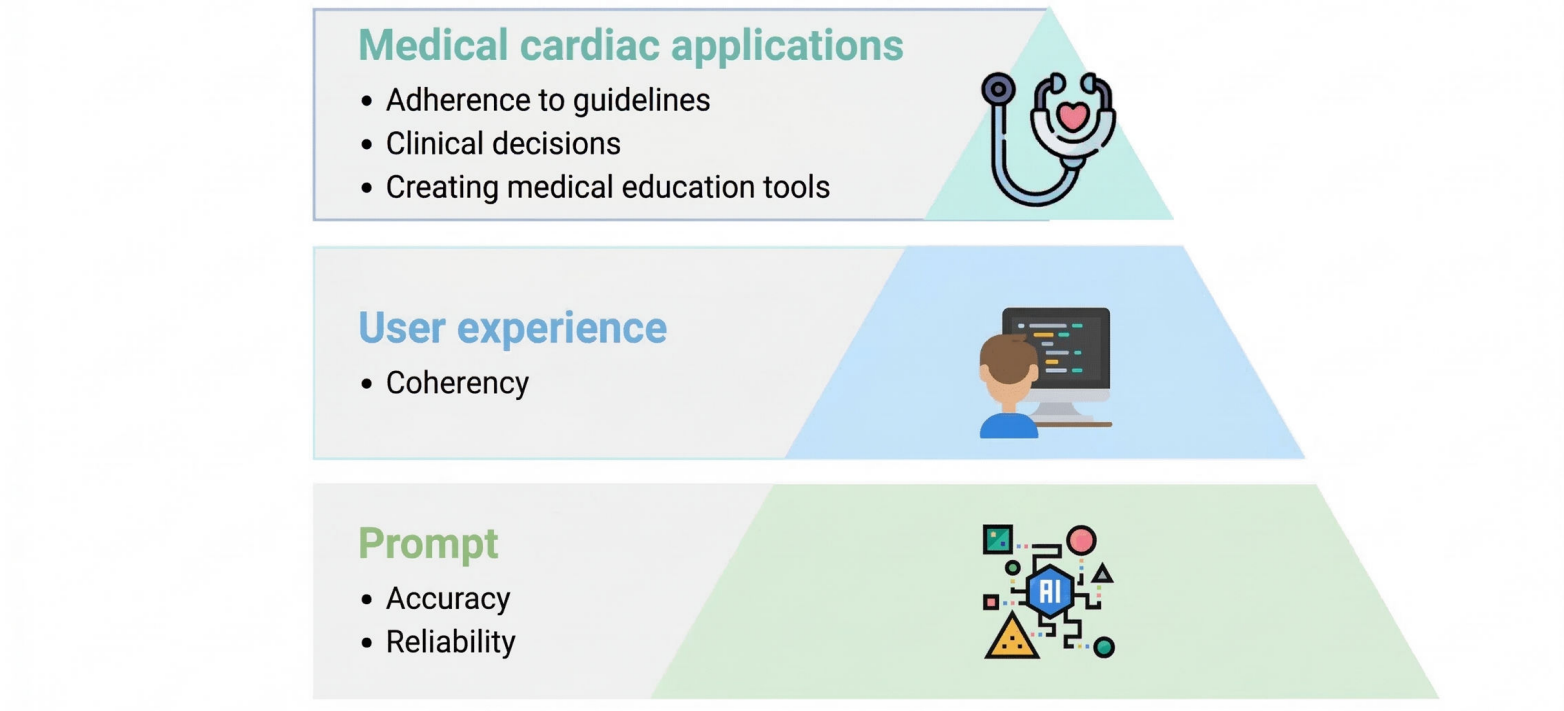
Illustration of 3 key focus areas regarding large language models in cardiology.

Figure 3 outlines the 3 primary aspects explored in the articles, including the reliability of LLMs, user interaction, and their specific applications in cardiology.

## Discussion

This systematic review included 35 studies [5,13-46] on the use of LLMs in cardiology, grouped into 5 domains: chronic and progressive cardiac conditions, acute cardiac events, physician education, patient education, and cardiac diagnostic tests. Overall, the studies showed that LLMs have potential across multiple aspects of cardiac management. In chronic conditions, models such as ChatGPT accurately answered common patient questions and improved the readability of educational materials [27], supporting patient engagement in long-term care. In acute emergencies, LLMs produced advice that users found clear, relevant, and accurate [38,39], suggesting possible value for lay and professional support in time-critical situations. For cardiac diagnostics, multimodal models performed well in ECG interpretation, often matching or surpassing human specialists [14], indicating potential to support clinical workflows and reduce routine workloads.

Despite these benefits, several issues must be addressed before LLMs can be used widely in cardiology. Accuracy and consistency varied significantly across models, with some producing unreliable or inconsistent interpretations [30,36], making their use in clinical settings uncertain. Readability and accessibility also remained challenging: although some LLMs improved clarity, others achieved lower reading levels only by removing essential information [33], raising concerns for patient communication across varying literacy levels. The use of LLMs also raises data privacy concerns [40], as their deployment requires strict protection of sensitive patient information.

The reviewed articles had several limitations. Approximately half the included studies evaluated ChatGPT-3.5, whose training data extended only to September 2021, limiting its ability to provide up-to-date information, an important consideration in cardiology. Most studies relied on expert evaluations rather than patient feedback, limiting insight into real-world usability. No included studies involved actual patients, raising questions about whether individuals can engage effectively with artificial intelligence–generated content. Regarding diagnostic applications, earlier studies often relied on text-based representations of multimodal data, such as written ECG or echocardiography descriptions, which may not fully capture real-world diagnostic complexity. However, recent studies have begun directly evaluating image-based analysis using multimodal models, including assessments of ECG image interpretation by ChatGPT-4–based systems, as demonstrated in a study by King et al [14]. Despite these advances, the current evidence remains limited in scope, and performance across multimodal tasks is heterogeneous, underscoring the need for larger, standardized evaluations of direct image analysis in cardiology.

This review also has its own limitations. First, the search was restricted to PubMed and Scopus, potentially missing studies in databases such as Embase or IEEE Xplore. Second, the inclusion criteria limited the review to peer-reviewed publications, excluding conference papers and preprint repositories such as arXiv and medRxiv, where important AI-related findings are often shared prior to peer review. Third, most included articles were in silico evaluations rather than prospective trials, limiting the generalizability of the findings. The heterogeneity of tasks and methods prevented meta-analysis. Fourth, due to the rapid evolution of LLMs and changing model nomenclature, our search strategy did not incorporate newer terms such as “Copilot” or broader descriptors such as “Generative AI,” which may have resulted in missing recently published studies. Finally, because technological advancements occur quickly, relevant studies and newer LLM applications may have emerged after the search was completed.

Across studies, the adapted QUADAS (by Whiting et al at the University of Bristol) assessment revealed methodological limitations specific to LLM research. Patient selection frequently presented a high risk of bias, as many studies used researcher-generated prompts or unvalidated questions without clinician or patient confirmation. In physician education studies, question banks commonly excluded media-based content such as ECGs or echocardiography clips, restricting the range of assessed cardiology skills. The index test domain was also often high risk, as most studies used single, nonreplicated runs without reporting temperature settings or model versioning. Reference standards were generally low risk, whereas flow, timing, and data management were limited by missing metadata, insufficient prompt transparency, and lack of full output logs.

Future research should address these limitations by conducting prospective clinical trials evaluating LLMs in real-world workflows, developing standardized metrics for accuracy, readability, and safety, and exploring electronic health record integration. Studies should also expand multimodal applications, including direct analysis of ECGs and imaging.

In conclusion, LLMs demonstrate potential in cardiology, particularly in educational applications and routine diagnostics. However, performance remains inconsistent across clinical scenarios, especially in acute care, where precision is critical. With continued refinement and responsible integration, LLMs may ultimately become valuable partners in cardiovascular care and help redefine what is possible in modern medicine.

## Supplementary material

10.2196/76734 Multimedia Appendix 1 Study characteristics outcomes of large language model applications, literature search strategy, and QUADAS-2 risk of bias assessment.

10.2196/76734 Checklist 1 PRISMA 2020 checklist.

## Abbreviations

ACLS: Advanced Cardiovascular Life Support
AED: Automated External Defibrillator
AHA: American Heart Association
BLS: Basic Life Support
CPR: cardiopulmonary resuscitation
ECG: electrocardiogram
FRE: Flesch Reading Ease
HEART: History, ECG, Age, Risk factors, Troponin risk algorithm
JSH: Japanese Society of Hypertension
LLM: large language model
MeSH: Medical Subject Headings
MKSAP-19: Medical Knowledge Self-Assessment Program, 19th edition
PALS: Pediatric Advanced Life Support
PRISMA: Preferred Reporting Items for Systematic Reviews and Meta-Analyses
