# Supplementary material for: Large Language Models in Cardiology: Systematic Review
Source: JMIR Cardio. 2026 Apr 16;10:e76734. doi: 10.2196/76734 (PMC13085985; doi:10.2196/76734)
Supplement: Multimedia Appendix 1 [file cardio-v10-e76734-s001.docx]

**Multimedia Appendix 1: Literature search strategy**

Database:

### **PubMed**, In-Process & Other Non-Indexed Citations and Daily <1946 to April 14, 2024>

1. ("Cardiovascular Diseases"[MeSH] OR "Heart Diseases"[MeSH] OR "Heart Failure"[MeSH] OR "Arrhythmias, Cardiac"[MeSH] OR "Myocardial Ischemia"[MeSH] OR "Acute Coronary Syndrome"[MeSH] OR "Heart Valve Diseases"[MeSH] OR "Echocardiography"[MeSH] OR "Electrocardiography"[MeSH] OR cardiology OR "heart failure" OR arrhythmia OR "atrial fibrillation" OR "myocardial infarction" OR "acute coronary syndrome" OR echocardiography OR electrocardiography OR ECG OR EKG OR "aortic stenosis")
2. ("Large Language Model" OR LLM OR LLMs OR ChatGPT OR "GPT-3" OR "GPT-4" OR Bard OR Gemini OR Claude OR LLaMA OR "Transformer-based model" OR OpenAI OR "Microsoft Bing" OR "Google Bard" OR "Google Gemini")
3. 1 AND 2

### **Scopus** (last searched April 14, 2024)

TITLE-ABS-KEY(
 ("cardiology" OR "cardiovascular" OR "heart failure" OR "arrhythmia" OR "atrial fibrillation"
 OR "myocardial infarction" OR "acute coronary syndrome" OR echocardiography
 OR electrocardiography OR ECG OR EKG OR "aortic stenosis"))

AND

TITLE-ABS-KEY(
 ("large language model" OR LLM OR LLMs OR ChatGPT OR "GPT-3" OR "GPT-4"
 OR "Google Bard" OR Gemini OR Claude OR LLaMA OR "Transformer-based model" OR OpenAI OR "Microsoft Bing"))

**Table S1:** Study characteristics

| Category | Authors | Dataset and Size (n) | Validation and Validation metrics | Reference for validation |
| --- | --- | --- | --- | --- |
| Chronic and Progressive Cardiac Conditions | [Dimitriadis, Fotis et al.](https://pubmed.ncbi.nlm.nih.gov/38451243/)  [27] | Queries knowledge and management of HF (47) | 2 cardiologists | ESC guidelines for the diagnosis and Treatment of acute and chronic heart failure |
|  | [Riddell, Craig W et al.](https://pubmed.ncbi.nlm.nih.gov/37964183/)  [33] | Queries knowledge and management of HF (16) | Scoring readability by FRE and SMOG | AHA and ECS |
|  | [Krittanawong, Chayakrit et al.](https://pubmed.ncbi.nlm.nih.gov/37832625/)  [34] | Inquiries from patients regarding HF and the cardiology clinic (20) | Cardiologists and HF specialists  Ranking -  “reliable but needs explanation”, “reliable without explanation”, or “unreliable” |  |
|  | [Rouhi, Armaun D et al.](https://pubmed.ncbi.nlm.nih.gov/38194058/)  [35] | Patient education materials on AS (21) | FRE, FKGL, GFI, SMOG Each score indicates the number of years of education necessary to understand the assessed reading material | Professional cardiothoracic surgical society and academic institutions in the USA |
|  | [Hillmann, Henrike A K et al.](https://pubmed.ncbi.nlm.nih.gov/38127304/)  [36] | Typical inquiries from patients regarding AF (25) or CIED (25) | 3 independent electrophysiologists, with a 4 reviewer for disagreement  WoC and FRE score |  |
|  | [Van Bulck, Liesbet, & Philip Moons](https://pubmed.ncbi.nlm.nih.gov/37094282/) [37] | Typical inquiries from virtual patients regarding CHD, AF, HF and Chol | 20 (19 nurses, 1 dietician)  Rating the case on a scale from 1 to 10.  Comparing the valuability to Google searching |  |
|  | [Kassab, Joseph et al.](https://pubmed.ncbi.nlm.nih.gov/37982246/)  [25] | 15 patient centered and 15 physician centered VHD queries | 3 structural cardiologists from Cleveland clinic (blinded) |  |
|  | [Han, Changho et al.](https://pubmed.ncbi.nlm.nih.gov/38357664/) [24] | UK Biobank (n=47,468); KoGES (5,718) | Comparison with ACC/AHA & Framingham  AUROC, AUPRC | ACC/AHA  Framingham |
|  | [Ali, Mohammed M et al.](https://pubmed.ncbi.nlm.nih.gov/38138852/) [23] | US county level datasets (CDC WONDER, US Census, USDA)  (3,118) | **Multivariate linear regression, cross validation, significance testing, R² analysis, β-coefficient evaluation,** and **consistency checks with epidemiological trends** across all counties | US national data sources |
|  | [Li, Pengfei et al.](https://pubmed.ncbi.nlm.nih.gov/38497458/) [22] | 25 ESC guideline queries | 2 physicians  Good / borderline / poor | ESC 2022 cardio oncology guideline |
|  | [Yano, Yuichiro et al.](https://pubmed.ncbi.nlm.nih.gov/37916418/)  [21] | 20 FAQs | 3 hypertension/nephrology specialists  Appropriate or inappropriate  Gwet’s agreement |  |
|  | Kusunose, Kenya et al.  [46] | A total of 31 JSH 2019 questions (clinical questions with subquestions + limited evidence based questions) | Judgement against guideline answers by a certified cardiology doctor.  χ² comparison of proportions, Shannon entropy | JSH 2019 guideline answer key |
|  | Al Tibi, Ghaith et al.  [45] | 40 patients clinical data | Cardiologist’s recommendations from the most recent lab review visit.  Match rate and Cohen’s kappa |  |
| Acute Cardiac Events | [Birkun & Gautam](https://pubmed.ncbi.nlm.nih.gov/37640177/) [38] | 60 prompts (20 each: Gambia, India, USA) | Guidelines Checklist  True/Partly/Not true; readability grade; superfluous directive rate | Educational guidelines 2020, Resuscitation, first aid guidelines |
|  | [Scquizzato, Tommaso et al.](https://pubmed.ncbi.nlm.nih.gov/38081504/)  [39] | 40 questions | 14 professionals and 16 laypeople  Ranking 1 (poor) to 5 (excellent) each feature examined | Sudden Cardiac Arrest UK focusing cardiac arrest and CPR |
|  | [Safranek, Conrad W et al.](https://pubmed.ncbi.nlm.nih.gov/38481520/)  [40] | 24 Medical notes of patients | Comparison of researchers calculation.  Non numeric response rate; mean subscore error; correct risk bucket % | HEART score components (History, ECG, Age, Risk factors, Troponin) |
|  | Birkun, Alexei  [43] | 20 Repeated standardized conversations  Scenario 1 “not breathing” n=10 Scenario 2 “not sure” n=10 | Checklist based assessment of dialogue components | AHA CPR guidelines and T-CPR protocols/public resources |
| Physician Education | [Harskamp, Ralf E, & Lukas De Clercq.](https://pubmed.ncbi.nlm.nih.gov/38348835/) [41] | 50 Multiple questions 20 Clinical cases | Cardiologist consensus | ESC guideline |
|  | [Skalidis, Ioannis et al.](https://pubmed.ncbi.nlm.nih.gov/37265864/)  [16] | 362 sample MCQs | Gold standard answers  % correct | ESC question sources, BHDRA questions and StudyPRN questions |
|  | [Yavuz, Y. E., & Kahraman, F](https://pubmed.ncbi.nlm.nih.gov/39049771/) [15] | 20 case vignettes | 18 cardiologists |  |
|  | [Gritti, Michael N et al.](https://pubmed.ncbi.nlm.nih.gov/38170274/)  [13] | 88 subspecialty questions | Official answer key of the textbook  % correct | Pediatric cardiology Board Review textbook |
|  | [Lee, Paul C et al](https://pubmed.ncbi.nlm.nih.gov/38938709/).  [5] | 120 Clinical cases | Physicians  % correct, 50% passing threshold | MKSAP-19 |
| Patient Education | Günay, Serkan et al.  [19] | 6 representative questions | 4 emergency medicine specialists  7 point Likert; RFE |  |
|  | [Bushuven, Stefan et al.](https://pubmed.ncbi.nlm.nih.gov/37987870/)  [20] | 22 Simulated pediatric scenarios,  3 times per model (132) | pediatric resuscitation specialists  Checklist guidelines | AHA/PALS guidelines |
|  | [Lautrup, Anton Danholt et al.](https://pubmed.ncbi.nlm.nih.gov/37945282/)  [18] | 123 prompts across languages and literacy levels | Expert review  4C scores (1–5 scale) |  |
|  | [Moons, Philip, and Liesbet Van Bulck](https://pubmed.ncbi.nlm.nih.gov/37603843/) [17] | 3 patient info texts | Readability formulas  FKGL, SMOG, FRE, GFI | Original published patient education materials |
|  | Almagazzachi, Ahmed et al.  [44] | 100 Curated hypertension FAQs; each asked three times | Clinical appropriateness vs guidelines and physician review; reproducibility defined as semantic consistency across runs | A composite of established hypertension reference publications |
| Cardiac Diagnostics Tests | [Fijačko, Nino et al.](https://pubmed.ncbi.nlm.nih.gov/37884222/)  [30] | 81 Multiple questions (27 for each Chatbot) | AHA ACLS exam answers  % correct | AHA,BLS and ACLS |
|  | Zhu, Lingxuan et al.  [42] | 75 official questions | AHA ACLS exam answers  % correct | AHA,BLS and ACLS |
|  | [King, Ryan C et al.](https://pubmed.ncbi.nlm.nih.gov/38160904/) [14] | 75 official questions | BLS and ACLS exam answers  % correct | BLS 2016 and ACLS exams |
|  | [Günay, Serkan et al.](https://pubmed.ncbi.nlm.nih.gov/38507847/)  [29] | 40 Multiple questions with ECG cases | 12 emergency medicine specialists and 12 cardiology specialist | “150 ECG Cases” book |
|  | [Kangiszer, Gyula et al.](https://pubmed.ncbi.nlm.nih.gov/37943230/)  [28] | 150 Multiple questions Open ended questions | Answers of Klein’s book  Comparison (1 for correct, 2 for incorrect) | Klein’s Clinical Echocardiography Review:  A Self Assessment Tool textbook |
|  | [Sarangi, Pradosh Kumar et al.](https://pubmed.ncbi.nlm.nih.gov/38549881/)  [26] | 15 cases (5 cardiovascular, 10 thoracic imaging patterns)  Total of 75 DDx | 2 cardiologists radiologists  Identification of top 5 DDx |  |

Abbreviations:

Clinical conditions and terms:

HF, heart failure; HFrEF, heart failure with reduced ejection fraction; AS, aortic stenosis; AF, atrial fibrillation; CIED, cardiac implantable electronic device; CHD, chronic heart disease; Chol, cholesterol; VHD, valvular heart disease; CVD, Cardiovascular Disease; MI, myocardial infarction; PAD, peripheral arterial disease; VV, varicose veins; CP, cardiovascular prevention; CPR, cardiopulmonary resuscitation; T-CPR, telecommunicator-assisted cardiopulmonary resuscitation; ECG, Electrocardiogram; DDx, differential diagnosis.

Guidelines, exams, and organizations:

ESC, European Society of Cardiology; AHA, American Heart Association; ACC, American College of Cardiology; JSH, Japanese Society of Hypertension; MCQs, multiple choice questions; PALS, Pediatric Advanced Life Support; BLS, Basic Life Support; ACLS, Advanced Cardiovascular Life Support; MKSAP-19, Medical Knowledge Self-Assessment Program (19th edition).

Datasets and registries:

KoGES, Korean Genome and Epidemiology Study; CDC WONDER, Centers for Disease Control and Prevention Wide ranging Online Data for Epidemiologic Research; USDA, United States Department of Agriculture; BHDRA, British Heart Data Research Alliance.

Readability and statistical metrics:

FRE, Flesch Reading Ease; SMOG, Simple Measure of Gobbledygook; FKGL, Flesch–Kincaid Grade Level; GFI, Gunning Fog Index; WoC, word count; AUROC, area under the receiver operating characteristic curve; AUPRC, area under the precision recall curve.

**Table S2:** Outcomes of large language model applications.

| Group | Manuscript | Qualitative | Quantitative |
| --- | --- | --- | --- |
| Chronic and Progressive Cardiac Conditions | [Dimitriadis, Fotis et al.](https://pubmed.ncbi.nlm.nih.gov/38451243/)  [27] | ChatGPT provided informative and supportive HF advice; generally accurate and patient friendly | ChatGPT-3.5 answered 43/47 (91%) HF patient questions adequately; 4/47 (9%) right but insufficient. |
|  | [Riddell, Craig W et al.](https://pubmed.ncbi.nlm.nih.gov/37964183/)  [33] | Answers were accurate and consistent but written at college reading level | ChatGPT-4 responses to 70 HF FAQs: 71% (50/70, median FRE 40.2 ≈ grade 16) at college level readability; 23% (16/70) at recommended lower than college level and 4% (4/70) of responses requiring grade 8-9. |
|  | [Krittanawong, Chayakrit et al.](https://pubmed.ncbi.nlm.nih.gov/37832625/)  [34] | Majority of HF answers considered reliable; some lacked depth or contained inaccuracies; highlighted need for clinician oversight | On 20 HF patient questions (200 responses), ChatGPT was reliable with explanation in 40% (8/20), reliable without explanation in 40% (8/20), and unreliable in 20% (4/20). |
|  | [Rouhi, Armaun D et al.](https://pubmed.ncbi.nlm.nih.gov/38194058/)  [35] | Both improved readability; ChatGPT better than Bard | ChatGPT-3.5 simplified aortic stenosis materials to 6th–7th grade, Bard to 8th–9th; both improved from college level baseline, all P<.001. |
|  | [Hillmann, Henrike A K et al.](https://pubmed.ncbi.nlm.nih.gov/38127304/)  [36] | ChatGPT-4 most accurate and comprehensible; Bard weakest; ChatGPT still omitted about quarter key content. | On 25 AF questions, ChatGPT-4 produced 84% (21/25) appropriate and 92% (23/25) comprehensible responses with 24% (6/25) missing content, outperforming Bing 60% (15/25), 88% (22/25) and 60% (15/25) and Bard 52% (13/25), 96% (24/25), 52% (13/25), respectively.  On 25 CIED questions, ChatGPT-4 produced 88% (22/25) appropriate and 100% (25/25) comprehensible responses with 52% (13/25) missing content, outperforming Bing 72% (18/25), 88% (22/25), 88% (22/25) and Bard 16% (4/25), 92% (23/25), 92% (23/25), respectively. |
|  | [Van Bulck, Liesbet, & Philip Moons](https://pubmed.ncbi.nlm.nih.gov/37094282/)  [37] |  | On four cardiology vignettes (CHD, AF, HF, cholesterol), 40% (8/20) of experts rated ChatGPT’s information as more valuable than Google, 45% (9/20) as equally valuable, and 15% (3/20) as less valuable. |
|  | [Kassab, Joseph et al.](https://pubmed.ncbi.nlm.nih.gov/37982246/)  [25] | ChatGPT-4 was 2.5 times more likely to provide an accurate answer compared with Google Bard (relative risk = 2.5; P< .0001). | On 30 valvular heart disease queries ChatGPT-4 provided 100% (15/15) accurate responses to patient centered questions and 73% (11/15) accurate with 27% (4/15) partly accurate responses to complex clinical scenarios, outperforming Google Bard (40% [6/15] accurate). |
|  | [Han, Changho et al.](https://pubmed.ncbi.nlm.nih.gov/38357664/)  [24] | ChatGPT-4 can estimate cardiovascular risk with accuracy similar to established prediction tools. | On 47,468 UK Biobank and 5,718 KoGES participants, ChatGPT-4 achieved AUROC 0.725 in the UKB and 0.664 in the KoGES cohort for 10 year CVD risk prediction, performing comparably to the ACC/AHA (0.733, 0.674) and Framingham (0.728, 0.675) models. |
|  | [Ali, Mohammed M et al.](https://pubmed.ncbi.nlm.nih.gov/38138852/)  [23] | Used for variable selection and regression; showed digital literacy and social vulnerability linked to CV mortality; ecological level only | On 3,118 U.S counties ChatGPT-4–assisted regression explained 34% (R² = 0.34) of the variability in age adjusted cardiovascular mortality, with higher social vulnerability increasing mortality (β = +49.01) and greater digital literacy reducing it (β = –4.51). |
|  | [Li, Pengfei et al.](https://pubmed.ncbi.nlm.nih.gov/38497458/)  [22] | ChatGPT-4 most accurate across domains; strong in definition/diagnosis, weaker in treatment/prevention due to outdated training | On 25 cardio oncology questions, ChatGPT-4 provided 68% (17/25) appropriate responses, followed by Bard, Claude 2, and ChatGPT-3.5 with 52% (13/25), and Llama 2 with 48% (12/25) (P =.653). |
|  | [Yano, Yuichiro et al.](https://pubmed.ncbi.nlm.nih.gov/37916418/)  [21] | Overall, ChatGPT-4 provided accurate, guideline consistent answers in both languages, though English outputs were more detailed and contextually comprehensive. | On 20 questions, ChatGPT-4’s responses were rated appropriate in 85% (17/20) of cases, with strong inter reviewer agreement (Gwet’s AC = 0.890, SE 0.066, P <.0001). Reviewers found the English responses superior in 11/20 questions, while Japanese responses were preferred in one. |
|  | Kusunose, Kenya et al.  [46] |  | Overall accuracy 64.5% (20/31). Accuracy was higher for clinical questions (CQs) than for limited evidence-based questions: 80% (16/20) vs 36% (4/11) (P=0.005). A nonsignificant trend was observed for recommendation-level versus evidence-level questions: 62% vs 38% (denominators not reported; P=0.070). No difference was found between questions originally written in Japanese and translated questions: 65% vs 58% (denominators not reported; P=0.602). Across 21 CQs, 9 showed zero entropy (identical answers), while 7 of the remaining 12 had entropy >0.5 (unacceptable variability) |
|  | Al Tibi, Ghaith et al.  [45] |  | Overall recommendations conflicted in **95% (38/40); Cohen’s kappa = −0.0127** (no agreement). Category match: **stop 0%, decrease 0%, increase 6.7% (3/40), add 12.5% (5/40)** |
| Acute Cardiac Events | [Birkun & Gautam](https://pubmed.ncbi.nlm.nih.gov/37640177/)  [38] | Country specific emergency numbers were incorrect in all of responses for India and the Gambia. | On 60 Bing chatbot responses, inconsistent advice appeared in 25% (5/20) of responses for the Gambia and USA and 45% (9/20) for India. Readability required a 12th grade level for the Gambia and USA and 10th grade for India (P ≤ .008). |
|  | [Scquizzato, Tommaso et al.](https://pubmed.ncbi.nlm.nih.gov/38081504/)  [39] | CPR related responses were rated lower across all parameters by both professionals and laypeople. | On 40 FAQs, ChatGPT-3.5 answers to cardiac arrest and CPR questions were rated positively overall (mean 4.3/5 ± 0.7), with high scores for clarity (4.4/5 ± 0.6), relevance (4.3/5 ± 0.6), and accuracy (4.0/5 ± 0.6). |
|  | [Safranek, Conrad W et al.](https://pubmed.ncbi.nlm.nih.gov/38481520/)  [40] | In both models there was a decrease in the rate of responses with erroneous, non numerical subscore answers. | On 1,200 prompt trials per model, ChatGPT-4 reduced non numerical errors from 5.7% (95% CI 3.6–8.9%) to 0.3% (0.1–1.9%), lowered subscore error to 0.10 (0.07–0.14) points with less variability (SD 0.33), and correctly classified HEART risk groups in 100% (96.3–100%) of runs, compared with 81.5% (71.7–88.4%) for ChatGPT-3.5. |
|  | Birkun, Alexei  [43] | The chatbot’s performance varied across cases, and key elements of bystander interrogation and CPR guidance were intermittently omitted. It asked only whether the victim was “breathing” rather than “breathing normally,” which could miss agonal breathing and delay recognition of cardiac arrest. The chatbot also did not inquire about nearby AED availability. | In Scenario 1, the chatbot suggested inapplicable/excessive actions in 10% (1/10) of conversations; in Scenario 2, this occurred in 30% (3/10).  In Scenario 2, the chatbot did not ask for the emergency address in 50% (5/10) of cases and failed to transition to CPR instructions after assessing the victim’s condition in 30% (3/10). |
| Physician Education | [Harskamp, Ralf E, & Lukas De Clercq.](https://pubmed.ncbi.nlm.nih.gov/38348835/)  [41] |  | The January 2023 version of ChatGPT-3.5 performed significantly worse, answering 74% (37/50) vs 92% (46/50) of trivia questions compared with the September 2023 ChatGPT version (P = .031), and only 50% (10/20) of complex cases correctly. |
|  | [Skalidis, Ioannis et al.](https://pubmed.ncbi.nlm.nih.gov/37265864/)  [16] | Showed ChatGPT could achieve passing level knowledge | On 362 Cardiology questions, ChatGPT answered 58.8% (213/362) correctly. ESC 61.7% (42/68), BHDRA 52.6% (79/150), StudyPRN 63.8% (92/144), approximating the 60% passing threshold. |
|  | [Yavuz, Y. E., & Kahraman, F](https://pubmed.ncbi.nlm.nih.gov/39049771/)  [15] |  | On 20 cardiology vignettes, ChatGPT-4 received high expert agreement for differential diagnoses (median 5, IQR 1) and management plans (median 4, IQR 1), with diagnostic accuracy of 4.47 ± 0.81 in Group 1 and 4.58 ± 0.67 in Group 2, with no significant difference between groups (P < .256). |
|  | [Gritti, Michael N et al.](https://pubmed.ncbi.nlm.nih.gov/38170274/)  [13] |  | On 88 pediatric cardiology knowledge questions, ChatGPT-4 answered 66% (58/88) correctly, significantly outperforming ChatGPT-3.5 at 38% c; p < .0001), with superior accuracy across every subspecialty topic. |
|  | [Lee, Paul C et al](https://pubmed.ncbi.nlm.nih.gov/38938709/).  [5] |  | On 120 cardiology questions, ChatGPT-4 outperformed average MKSAP-19 users 80% (96/120) vs 60% (72/120; P = .0004; ChatGPT-3.5 also passed but lower at 55% (66/120), while PubMedGPT failed at 27% (32/120). |
| Patient Education | Günay, Serkan et al.  [19] | ChatGPT-4 responses were rated comparable to hospital websites across scientific accuracy, ease of understanding, and overall satisfaction, with no significant differences between groups (all P > .05). | Readability analysis showed: hospital website answers averaged a Flesch Reading Ease score of 65.6 (7th grade level), whereas ChatGPT-4 responses averaged 43.3 (11th grade level). |
|  | [Bushuven, Stefan et al.](https://pubmed.ncbi.nlm.nih.gov/37987870/)  [20] | ChatGPT-4 showed higher consistency than ChatGPT (Fleiss’ κ = 0.73 vs 0.48 for first aid advice), indicating more reliable performance across repeated scenarios. | On 22 pediatric emergency cases ChatGPT-3.5 and ChatGPT-4 correctly identified the diagnosis in 94% (124/132; P =.49) of responses, but advised emergency calls in only 54% (12/22) and provided correct first-aid guidance in 45% (10/22), with incorrect advanced life support instructions in 14% (3/22) of cases. |
|  | [Lautrup, Anton Danholt et al.](https://pubmed.ncbi.nlm.nih.gov/37945282/)  [18] | Higher literacy prompts produced better answers, and unexpectedly, responses in the lower resource language were often of higher quality. | ChatGPT-4 responses to 123 cardiovascular prompts averaged 3–4 across the 4Cs (correctness 3.45/5 conciseness 3.20/5, comprehensiveness 3.52/5, comprehensibility 3.72/5). Myocardial infarction prompts scored highest (correctness 3.84/5; conciseness 3.65/5), while cardiovascular prevention scored lowest (correctness 3.03/5; conciseness 2.71/5). |
|  | [Moons, Philip, and Liesbet Van Bulck](https://pubmed.ncbi.nlm.nih.gov/37603843/)  [17] |  | ChatGPT lowered readability modestly (JAMA grade 11→9; Cochrane 17→11; EJCN grade 10 unchanged) while preserving most content, with word counts changing minimally in JAMA (533→525), by 14% in Cochrane (365→315), and by 45% in EJCN (1,013→563). In contrast, Bard reached lower grade levels but removed substantial content, shortening the texts by 61% (525→207), 34% (365→242), and 80% (1,013→204), resulting in major information loss. |
|  | Almagazzachi, Ahmed et al.  [44] |  | Appropriateness: 93% (93/100) overall and 7% (7/100) inappropriate, evaluated against guideline-based standards. Reproducibility: 93% (93/100) of questions reproducible and 7% (7/100) irreproducible. |
| Cardiac Diagnostics Tests | [Fijačko, Nino et al.](https://pubmed.ncbi.nlm.nih.gov/37884222/)  [30] | ChatGPT-4 Pro best, always answered; Bard moderate; Bing poor | On 27 ECG image interpretations, ChatGPT-4 was correct in 17/27 (63%), Bard 13/27 (48.2%), Bing 6/27 (22.2%). |
|  | Zhu, Lingxuan et al.  [42] | 75 item AHA BLS/ACLS exam (25 BLS and 50 ACLS) were evaluated, with 38 ACLS questions analyzable due to image processing constraints. | ChatGPT achieved 84% (21/25) overall accuracy on BLS and 78.9% (30/38) on evaluable ACLS items using multiple choice inputs, improving to 96% (24/25) and 92.1% (35/38) when incorrectly answered questions were rewritten as open ended prompts. |
|  | [King, Ryan C et al.](https://pubmed.ncbi.nlm.nih.gov/38160904/)  [14] |  | ChatGPT-4V answered 96%(24/25) BLS and 90% (45/50) ACLS questions correctly, accuracy decreased to 75% (9/12) for questions containing ECG. |
|  | [Günay, Serkan et al.](https://pubmed.ncbi.nlm.nih.gov/38507847/)  [29] | For everyday ECGs, ChatGPT-4 performed significantly better than both groups (P < .001 and P = .001), while in more challenging cases it surpassed emergency physicians (P <.001) but did not differ significantly from cardiologists (P = .190). | Across 40 ECG vignettes, ChatGPT-4 correctly answered 91% (36/40), outperforming emergency medicine specialists 77% (31/40, P < .001) and cardiologists 82% (33/40, P = .001). |
|  | [Kangiszer, Gyula et al.](https://pubmed.ncbi.nlm.nih.gov/37943230/)  [28] | Fact based questions making up the majority of correct responses across formats. | ChatGPT-4 answered 47% (67/141) in open ended format, 53% (75/141) in multiple choice without justification, and 55% (78/141) in multiple choice with justification formats correctly. |
|  | Sarangi, Pradosh Kumar et al. [26] |  | Across 15 cardiothoracic imaging patterns (75 total differentials), Perplexity performed highest with 67% (50/75) concordance, followed by ChatGPT at 65% (49/75) and Bing at 63% (47/75), while Bard showed the lowest performance at 45% (34/75). |

**Table S3:** Quality Assessment of Diagnostic Accuracy Studies-2 (QUADAS-2) risk of bias assessment.

Abbreviations: Pt. patient; Ref. reference.
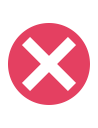
 = high risk of bias;
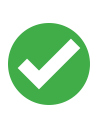
 = low risk of bias.

^a^ Insufficient description of the dataset or prompt selection process; ^b^ Lack of external validation or testing beyond the development dataset; ^c^ Unclear who performed the evaluation or grading of LLM outputs; ^d^ Inconsistent evaluation procedure across prompts or models; ^e^ Failure to specify ethical approval or data-source transparency where applicable.

|  | | **Risk of bias** | | | | | |
| --- | --- | --- | --- | --- | --- | --- | --- |
| **Author** | | **Pt. selection^a^** | | **Index test^b^** | **Ref. standard^c^** | **Flow and timing^d^** | **Data management^e^** |
| Dimitriadis, Fotis et al. [27] | | 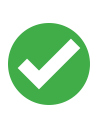 | | 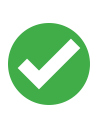 | 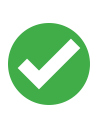 | 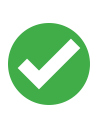 | 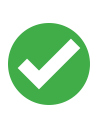 |
| Riddell, Craig W et al. [33] | | 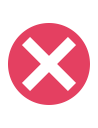 | | 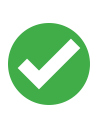 | 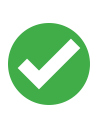 | 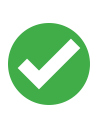 | 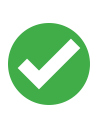 |
| Krittanawong, Chayakrit et al. [34] | | 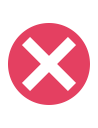 | | 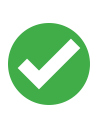 | 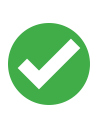 | 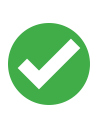 | 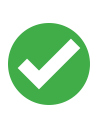 |
| Rouhi, Armaun D et al. [35] | | 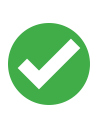 | | 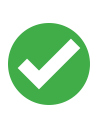 | 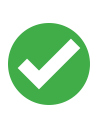 | 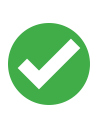 | 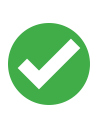 |
| Hillmann, Henrike A K et al. [36] | | 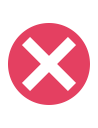 | | 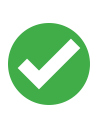 | 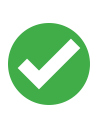 | 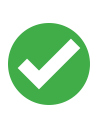 | 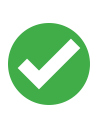 |
| Van Bulck, Liesbet, & Philip Moons [37] | | 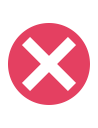 | | 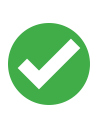 | 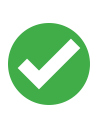 | 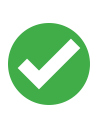 | 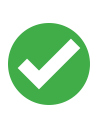 |
| Kassab, Joseph et al. [25] | | 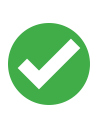 | | 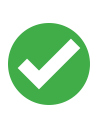 | 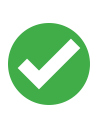 | 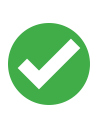 | 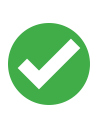 |
| Han, Changho et al. [24] | | 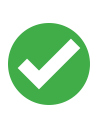 | | 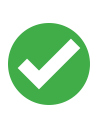 | 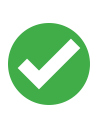 | 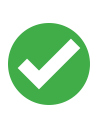 | 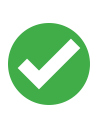 |
| Ali, Mohammed M et al. [23] | | 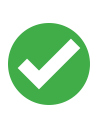 | | 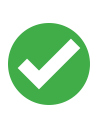 | 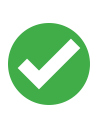 | 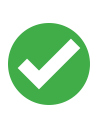 | 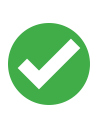 |
| Li, Pengfei et al. [22] | | 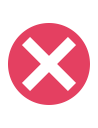 | | 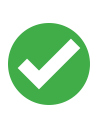 | 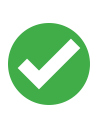 | 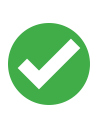 | 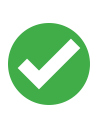 |
| Yano, Yuichiro et al. [21] | | 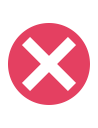 | | 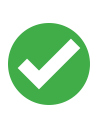 | 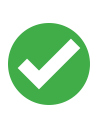 | 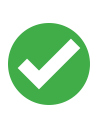 | 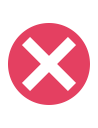 |
| Kusunose, Kenya et al. [46] | | 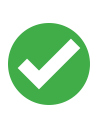 | | 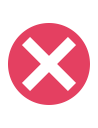 | 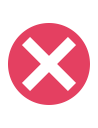 | 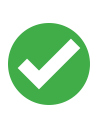 | 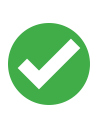 |
| Al Tibi, Ghaith et al. [45] | | 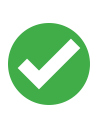 | | 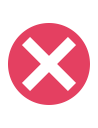 | 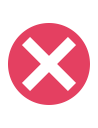 | 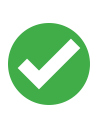 | 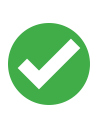 |
| Birkun, A. A., & Gautam, A. [38] | | 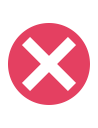 | | 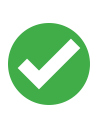 | 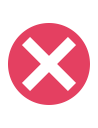 | 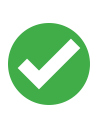 | 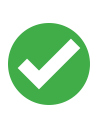 |
| Scquizzato, Tommaso et al. [39] | | 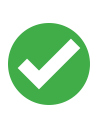 | | 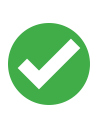 | 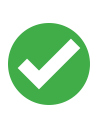 | 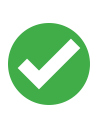 | 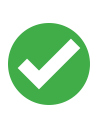 |
| Safranek, Conrad W et al. [40] | | 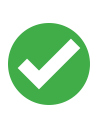 | | 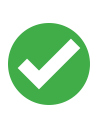 | 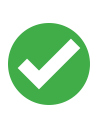 | 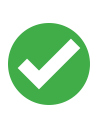 | 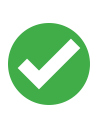 |
| Birkun, Alexei [43] | | 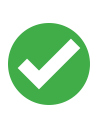 | | 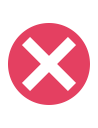 | 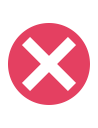 | 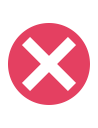 | 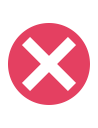 |
| Harskamp, Ralf E, & Lukas De Clercq. [41] | | 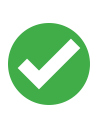 | | 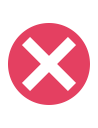 | 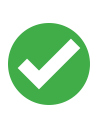 | 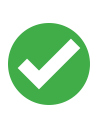 | 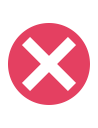 |
| Skalidis, Ioannis et al. [16] | | 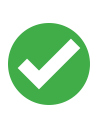 | | 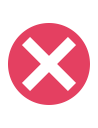 | 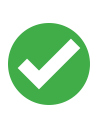 | 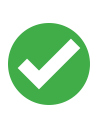 | 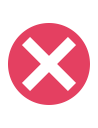 |
| Yavuz, Y. E., & Kahraman, F [15] | | 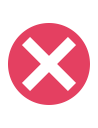 | | 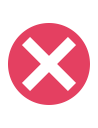 | 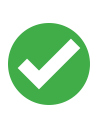 |  |  |
| [Gritti, Michael N et al.](https://pubmed.ncbi.nlm.nih.gov/38170274/) [13] | |  | |  |  |  |  |
| Lee, Paul C et al. [5] | |  | |  |  |  |  |
| Günay, Serkan et al. [19] | |  | |  |  |  |  |
| Bushuven, Stefan et al. [20] | |  | |  |  |  |  |
| [Lautrup, Anton Danholt et al.](https://pubmed.ncbi.nlm.nih.gov/37945282/) [18] | |  | |  |  |  |  |
| Moons, Philip, and Liesbet Van Bulck [17] | |  | |  |  |  |  |
| Almagazzachi, Ahmed et al.  [44] | |  | |  |  |  |  |
| Fijačko, Nino et al. [30] | |  | |  |  |  |  |
| Zhu, Lingxuan et al. [42] | |  | |  |  |  |  |
| King, Ryan C et al. [14] | |  | |  |  |  |  |
| [Günay, Serkan et al.](https://pubmed.ncbi.nlm.nih.gov/38507847/) [29] | |  | |  |  |  |  |
| Kangiszer, Gyula et al. [28] | |  | |  |  |  |  |
| Sarangi, Pradosh Kumar et al. [26] | |  | |  |  |  |  |
